# Costs and effectiveness of pharmacist-led group medical visits for type-2 diabetes: A multi-center randomized controlled trial

**DOI:** 10.1371/journal.pone.0195898

**Published:** 2018-04-19

**Authors:** Wen-Chih Wu, Tracey H. Taveira, Sean Jeffery, Lan Jiang, Lisa Tokuda, Joanna Musial, Lisa B. Cohen, Fred Uhrle

**Affiliations:** 1 Center of Innovation in Long-Term Services and Supports for Vulnerable Veterans, VA Medical Center, Providence, RI; 2 Department of Medicine, Brown University, Providence, RI; 3 University of Rhode Island College of Pharmacy, Kingston, RI; 4 University of Connecticut School of Pharmacy, Storrs, CT; 5 West Haven VA Medical Center, West Haven, CT; 6 VA Pacific Islands Health Care System, Honolulu, HI; TNO, NETHERLANDS

## Abstract

**Objectives:**

The effectiveness and costs associated with addition of pharmacist-led group medical visits to standard care for patients with Type-2 Diabetes Mellitus (T2DM) is unknown.

**Methods:**

Randomized-controlled-trial in three US Veteran Health Administration (VHA) Hospitals, where 250 patients with T2DM, HbA1c >7% and either hypertension, active smoking or hyperlipidemia were randomized to either (1) addition of pharmacist-led group-medical-visits or (2) standard care alone for 13 months. Group (4–6 patients) visits consisted of 2-hour, education and comprehensive medication management sessions once weekly for 4 weeks, followed by quarterly visits. Change from baseline in cardiovascular risk estimated by the UKPDS-risk-score, health-related quality-of-life (SF36v) and institutional healthcare costs were compared between study arms.

**Results:**

After 13 months, both groups had similar and significant improvements from baseline in UKPDS-risk-score (-0.02 ±0.09 and -0.04 ±0.09, group visit and standard care respectively, adjusted p<0.05 for both); however, there was no significant difference between the study arms (adjusted p = 0.45). There were no significant differences on improvement from baseline in A1c, systolic-blood-pressure, and LDL as well as health-related quality-of-life measures between the study arms. Compared to 13 months prior, the increase in per-person outpatient expenditure from baseline was significantly lower in the group visit versus the standard care arm, both during the study intervention period and at 13-months after study interventions. The overall VHA healthcare costs/person were comparable between the study arms during the study period (p = 0.15); then decreased by 6% for the group visit but increased by 13% for the standard care arm 13 months post-study (p<0.01).

**Conclusions:**

Addition of pharmacist-led group medical visits in T2DM achieved similar improvements from baseline in cardiovascular risk factors than usual care, but with reduction in the healthcare costs in the group visit arm 13 months after completion compared to the steady rise in cost for the usual care arm.

**Trial registration:**

NCT00554671 ClinicalTrials.gov

## Introduction

More than half of patients with diabetes die of cardiovascular disease [1, 2], which is largely preventable by simultaneous control of multiple cardiovascular risk factors [3–7].However, concomitant multi-factorial therapy using vigorous behavioral and pharmacologic interventions are labor intensive and costly [3, 8], and difficult to accomplish in traditional care settings [9–11]. Group medical visits have been proposed to be an efficient method to achieve cardiovascular risk reduction in diabetes mellitus (DM) and may lead to cost savings [12–15].

The roles of pharmacist’s participation in chronic disease care have continued to expand to meet growing health care demands in the United States [16]. After an initial diagnosis is made by the physician, pharmacists can function as health care providers through collaborative practice agreements to perform “comprehensive medication management [17].” This consists of clinical assessments and development of therapeutic plans, including but not restricted to prescriptive authority, ordering and interpretation of laboratory tests and care coordination, among other functions [16, 17]. Although single site studies have shown efficacy of pharmacist-led group medical visits in diabetes to improve glycemia and cardiovascular risk [18, 19], the *effectiveness* of such interventions across different sites is unknown. Moreover, experimental evidence is also lacking on how pharmacist-led group medical visits may improve health-related quality-of-life and institutional costs, both of which are needed for a widespread adoption of this method of “delivery” of care.

We conducted a 3-site randomized-controlled trial within the hospitals in the United States Veterans Health Administration (VHA) to determine the impact of a pharmacist-led group medical visit intervention added to standard care compared to standard care alone for patients with Type-2 DM, on glycemia, blood pressure, lipids, health status and healthcare system costs. We also explore the economic impact of this intervention 13 months after the conclusion of the intervention.

## Methods

### Design

This was a multisite, randomized, controlled, parallel design clinical trial involving three VHA Hospitals (ClinicalTrials.gov Identifier NCT00554671) (S1 Table). The Institutional Review Board and Research and Development Committees by the Providence VAMC, West Haven VAMC and Honolulu VAMC approved the protocol (S1 Text). Study procedures were conducted in accordance with the ethical standards of the Helsinki Declaration of 1975.

### Study sample

Inclusion criteria: All patients >18 years old with documented Type-2 DM in the medical record, last recorded hemoglobin A1c of >7.0% and at least one of the following: being a smoker (any cigarette smoking < 30 days), having an LDL >100 mg/dl in the last blood draw or a blood pressure >130/80 mm Hg documented on at least two occasions within the last 6 months, and able to participate in group discussions for diabetes and cardiovascular risk factors.

Patients were excluded if they had psychiatric instability (acutely suicidal, psychotic) or organic brain injury that precluded them from performing DM self-care. Patients with conditions that would preclude them from standard algorithm-based medication dose titrations such as pregnancy, complex co-morbidities as defined by New York Heart Association Class 3 or 4 heart failure, liver cirrhosis, end-stage renal disease on dialysis and end-stage cancer were also excluded from the study. Enrollment for this study began in July 2008 and study visits completed in June 2012, while economic cost data was collected for additional 13 months after study closure until July of 2013.

### Randomization

Participants were randomized using urn stratified randomization generated by an automated computer sequence based on the study sites (3 sites), number of uncontrolled cardiac risk factors (Blood Pressure >130/80 mmHg; or LDL-cholesterol >100mg/dL or active tobacco use within the previous 30 days) and previous participation in diabetes self-management (DSME) or VHA weight loss (Project MOVE!) programs to either (1) Group visits in addition to standard primary care or (2) standard primary care alone for 13 months.

#### Intervention

Group Medical Visits: Coordinated and led by clinical pharmacists, consisted of education on diabetes self-care during the first hour followed by behavioral counseling and comprehensive medication management for hyper- (and hypo-) glycemia, hypertension, and dyslipidemia during the second hour. The educational component included interactive lectures that were based on the American Diabetes Association (ADA) Standards of Diabetes Self-Management. Sessions were focused on one or two of the core diabetes content areas, such as healthy eating or physical activity, as identified by the National Diabetes Self-Management Education Task Force [17, 20]. Where appropriate, the one hour education was provided by a nutritionist, nurse or a physical therapist in separate sessions. The second hour comprehensive medication management consisted of clinical assessments and development of therapeutic plans, including algorithm-based medication initiation and management (guided by VHA Clinical Practice Guidelines)[21], ordering and interpretation of laboratory tests and care coordination by a clinical pharmacist [17]. The clinical pharmacists who delivered these group medical visits were required to have at least one-year of postgraduate residency training or equivalent in outpatient clinical care and/or were certified diabetes educators. Each group visit consisted of 4–6 participants and lasted 120 minutes. Group medical visits were held once weekly for over 4 weeks followed by 4 booster sessions held once every 3 months for a total of 13 months.

#### Standard care

Consisted of regular visits with primary care and specialty care providers at the VHA hospitals through individual contact. The average patient panel size is approximately 1,200 patients for each primary care provider. Patients with diabetes receive an average of 4 visits per year with their primary care provider with additional visits scheduled on an as needed basis determined per provider discretion. Performance of primary care providers in blood pressure, lipids and A1c is being periodically monitored and compared within and between the VHA hospitals by local and national VHA leadership.

### Outcomes

Primary outcomes: We study the modifiable cardiovascular risk factors of the UKPDS risk engine [22], a model for prediction of future coronary heart event risk in patients with Type-2 DM, which consisted of: change from baseline in A1c, systolic blood pressure and LDL-cholesterol at 13-months. The study intended to enroll 220 participants in 2-sites with a third site added 2 years after to increase ethnic diversity of the study population. The third site enrolled 30 patients following the current study protocol and was included in the analysis, and another 30 participants intended to pilot a video-conference approach of group medical visits and was not included in the current report. The 250 participants were powered to detect at 90% beta and 2-sided alpha of 0.05 to detect a 0.04 (SD 0.1) difference in UKPDS risk score between study arms using a two-sample T-test.

Secondary outcomes included change from baseline in health-related quality-of-life as measured by veteran version of Medical Outcomes Study survey (SF-36v)[23] and in healthcare costs from the payer (VHA costs) perspective. Healthcare costs from the VHA included provider time in the group medical visits (only for the intervention arm), medications, hospitalizations, Emergency Department visits, laboratory testing, procedures, outsource referral or transfer to non-VHA facilities paid by the VHA and outpatient clinic visits.

### Data collection

Patient comorbidities and demographics were collected directly from medical records based on ICD-9 codes and physician documentation in progress notes, and confirmed by interview. The primary (A1c, lipid profile, and systolic blood pressure) and secondary outcomes (SF-36v) were collected at baseline, 6-months and 13-months by a research assistant blinded to treatment arm allocations. Data collection for healthcare costs from the VHA perspective were gathered from a variety of VHA sources that included pharmacy/medication costs (DSS files) [24], the Fee basis files (use of non-VHA health services paid by the VHA), outpatient costs (urgent care, clinic visits, laboratory, and outpatient procedure costs) and the hospitalization-related costs from the VHA inpatient files. The cost of added pharmacist and other staff time (e.g. added 1h of nutritionist, 1h of nursing and 1h of physical therapist for each patient during the study period) was derived from an average of the participating VHA provider’s annual wage in the three sites. The staff time was calculated based on an average time spent by providers of each discipline of 3 participating sites in the group medical visits which were tracked quarterly using activity logs to include the time spent in the preparation of and during the group medical visits, phone follow-ups with the patients, and time spent in case discussions with other providers and chart documentation of the clinical encounters [25]. VHA costs were abstracted from VHA datasets for 13 months before, 13 months during and 13 months after the conclusion of study interventions.

### Statistical analysis

We conducted an intention-to-treat analysis. Baseline characteristics amongst the study arms were described. In order to compare the difference in outcomes over time (for within and between group comparisons), we used Generalized Estimating Equations (GEE) modeling to adjust for the randomization stratifiers[26] (site, number of uncontrolled cardiac risk factors and previous participation in diabetes self-management or weight loss programs) with a random effect of patients nested under VHA hospitals to account for repeated measures of the outcome. For the analysis of the healthcare cost data, cost was log-transformed to approximate normality. An average cost was determined for each patient for each of the three periods: pre-intervention (13 months before study interventions), intervention (during the 13 months of the randomized-controlled trial), and post-intervention (13 months after the randomized-controlled trial period of the study), and prorated for the days the patient was alive. Exploratory analysis was conducted to compare the change in VHA healthcare costs between the post-intervention period and the intervention period, and between the post-intervention period and the baseline pre-intervention period.

Sensitivity analysis of the cost was repeated using a subsample of patients propensity-matched (1:3 ratio) on the probability of receiving the study intervention and two strategies to negotiate missing cost data (imputation of median cost and deletion of the participant with missing observation).

All analyses were done on an intention-to-treat basis and performed using SAS 9.2 (Cary, NC). A 2-sided P value of 0.05 or less was considered to be significant.

## Results

### Study population and baseline characteristics

**Fig 1:**
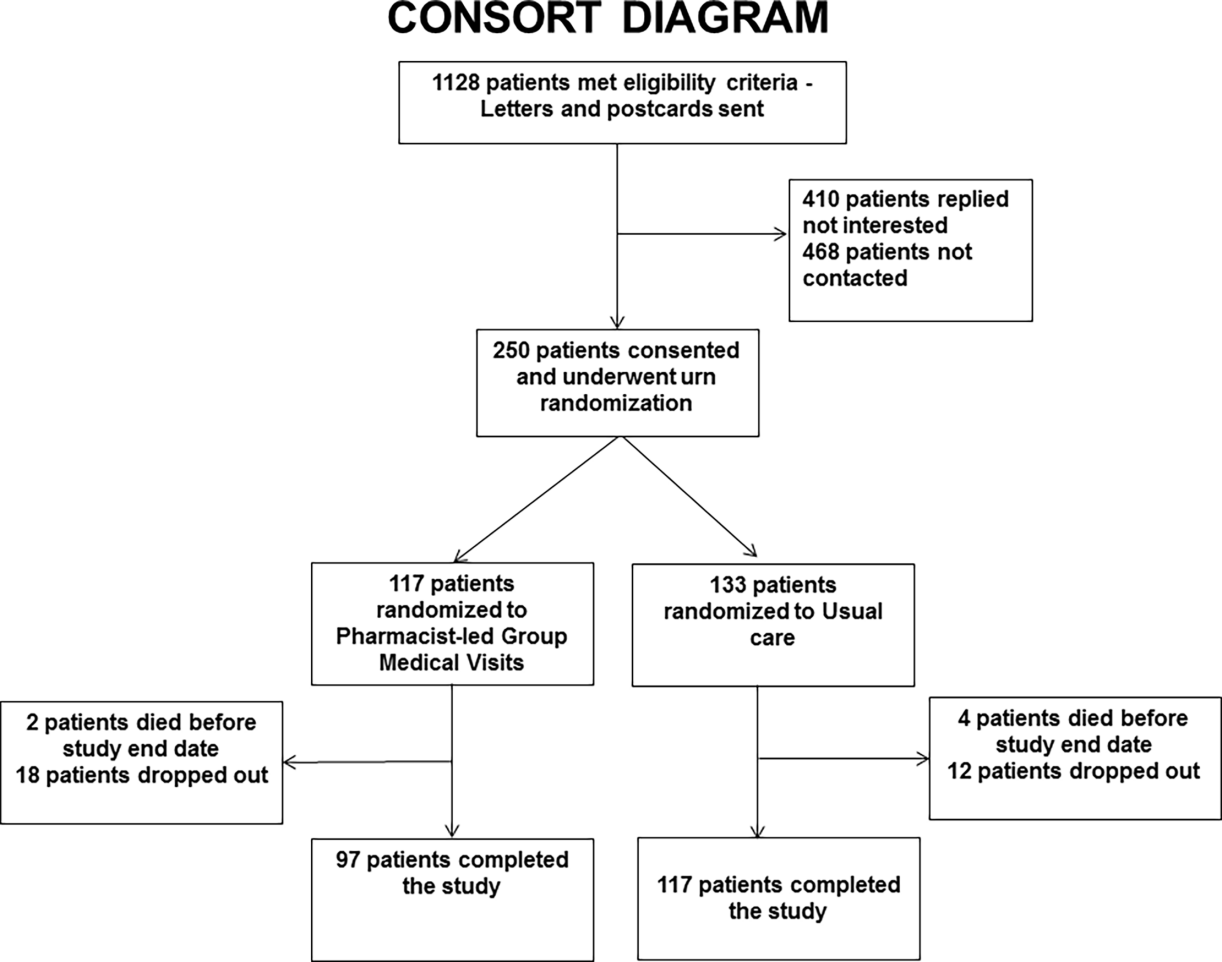
Consort diagram showing the enrollment, randomization and follow-up of study participants.

Of 1,128 patients who met eligibility criteria, 660 were contacted, and 250 patients (38%) were enrolled into the study (Fig 1). Based on cardiovascular risk factors, study site and previous participation in diabetes self-management programs, 117 patients were randomized to the group visit arm and 133 patients to standard care. Table 1 of baseline characteristics showed both group visit versus standard care arms to be comparable in demographic characteristics such as mean age (65.8 versus 65 years), gender (95.7 versus 96% male), and race (10 versus 11% African Americans). Clinical characteristics were also similar with the exception of previous stroke (10.3 versus 3.8%, average hospitalizations 13 months prior (0.4 versus 0.1), and total cholesterol levels (155 versus 165 mg/dL), for group visit and standard care arms, respectively.

**Table 1:** Baseline characteristics of study patients.

Mean ± SD unless otherwise indicated	Group Visits (n = 117)	Standard Care (n = 133)
Age (yr)	65.8 ± 8.7	65.0 ± 9.8
Male sex	95.7	96.2
Race		
• Caucasian (%)	75.0	78.2
• African American (%)	10.3	11.3
• Asian Pacific Islander (%)	12.9	6.0
• Hispanic (%)	0	3.0
• Unknown (%)	1.7	1.5
Duration of Diabetes (years)	13.5 ± 9.4	12.4 ± 9.9
Body mass index (Kg/m^2^)	32.8 ± 0.5	33.4 ± 0.6
Average number of hospitalizations 13 months prior to the study	0.4 ± 1	0.1 ± 0.4
Hypertension (%)	92.3	85.0
Hyperlipidemia (%)	87.2	91.0
Coronary artery disease (%)	39.3	43.6
Stroke (%)	10.3	3.8
Congestive heart failure (%)	10.3	9.8
COPD (%)	11.1	15.8
Anxiety disorder (%)	32.5	29.3
Mood disorder (%)	35.9	39.1
Schizophrenia/affective (%)	0.9	2.3
Nephropathy (%)	10.3	9.0
Neurophathy (%)	35.0	30.8
Retinopathy (%)	19.7	12.8
Active smoker (%)	21.4	27.1
Previous participation into DM self-care management programs (%)	27.0	20.0
Previous participation into VHA weight loss program (MOVE!) (%)	3.4	6.0
UKPDS 10-yr Coronary Risk	0.35 ± 0.19	0.36 ± 0.20
Systolic blood pressure (mmHg)	136.5 ± 19.0	136.2 ± 17.4
Diastolic blood pressure (mmHg)	75.1 ± 11.4	75.3 ± 10.5
Total Cholesterol (mg/dL)	155.2 ± 33.5	165.3 ± 39.7
HDL Cholesterol (mg/dL)	37.8 ± 10.9	38.1 ± 9.5
LDL Cholesterol (mg/dL)	87.6 ± 29.8	93.7 ± 33.7
Hemoglobin A1c (%)	8.2 ± 1.5	8.2 ± 1.3
Creatinine (mg/dL)	1.1 ± 0.4	1.1 ± 0.3
Microalbumin/creatinine ratio	198.4 ± 581.6	114.3 ± 267.4
SF-36v Physical component scale	39.2 ± 9.7	39.3 ± 9.8
SF-36v Mental component scale	48.8 ± 11.0	50.4±11.7
Healthcare Costs 13 months before the study, $	22062 ± 67524	15200 ± 23812

**SI conversion factors:** cholesterol mg/dL to mmol/L, multiply by 0.0259; creatinine mg/dL to umol/L, multiply by 88.4 . **Abbreviations:** COPD = chronic obstructive pulmonary disease, DM = Diabetes, UKPDS = United Kingdom Prospective Diabetes Study Coronary Event Risk score

### Participants and attendance

Over the 13 month study period, participants in the group visits arm attended 749 of the scheduled 920 sessions (81.4% attendance). Complete 13-month follow-up data was available on 97/117 participants (82.9%) randomized to the group visits arms and 117/133 (87.9%) participants in the standard care arm.

### UKPDS risk score, hemoglobin A1c, blood pressure and lipids

**Fig 2:**
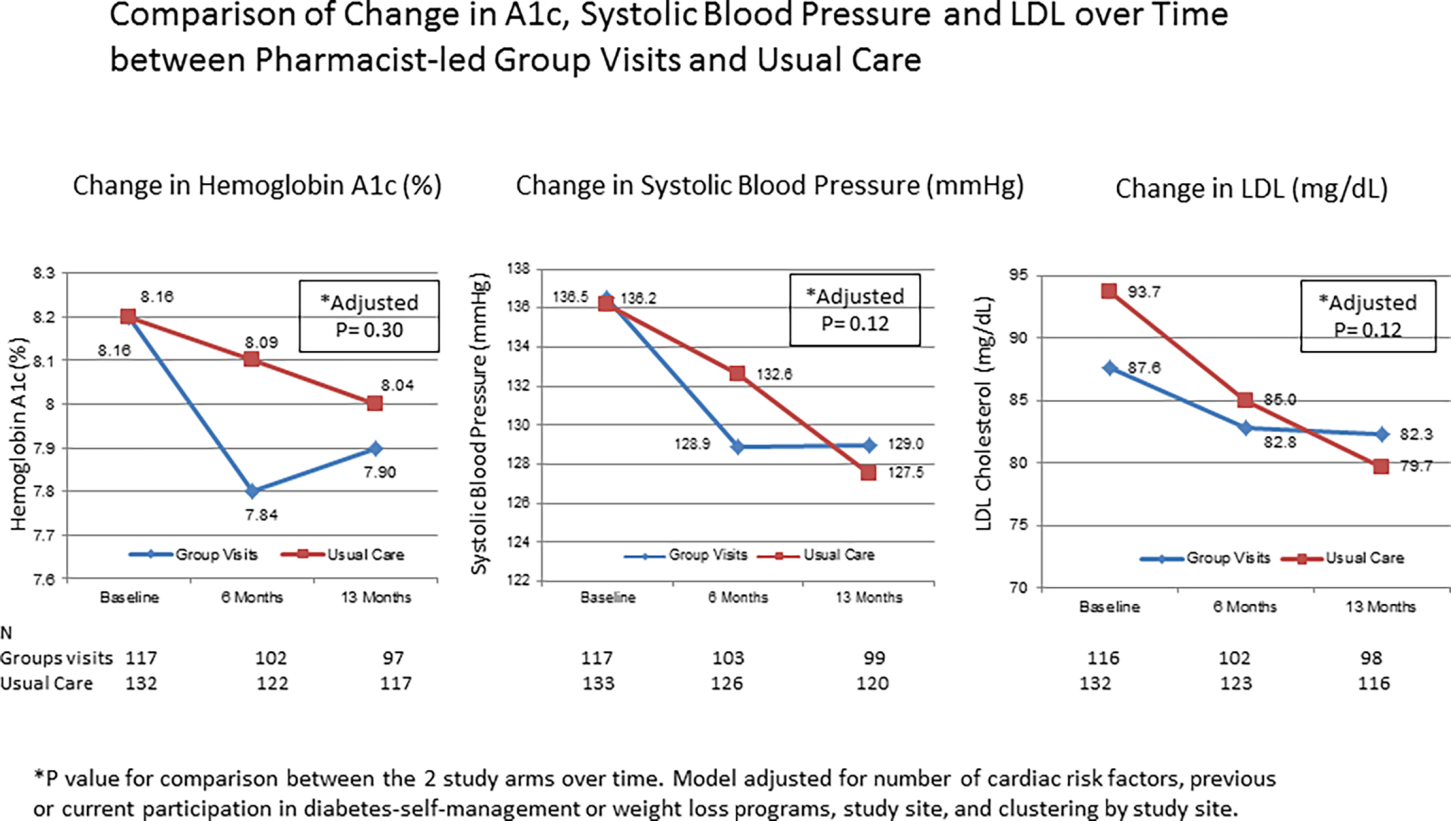
Change in hemoglobin A1c, blood pressure and lipids during the study period. There were no significant differences on improvement from baseline in A1c, systolic-blood-pressure, and LDL between group visits and standard care.

After 13 months of the trial, the mean change in the UKPDS coronary event risk was significantly reduced for patients in both arms (-0.02 ±0.09 and -0.04 ±0.09, group visit and standard care respectively, adjusted p<0.05 for both); however, there was no significant difference between the study arms (adjusted p = 0.45). There were no significant differences between the group visit and the standard care arms in the change from baseline in hemoglobin A1c (-0.27±1.25% versus -0.14±1.23%, adjusted p = 0.30), systolic blood pressure (-6.9±19.7 versus -8.9±17.4 mmHg, adjusted p = 0.12) or LDL levels (-5.4 ±30.1 versus -14.2±30.0 mg/dL, adjusted p = 0.12), respectively (Fig 2).

### Health status by medical outcomes study survey (SF-36v)

**Fig 3:**
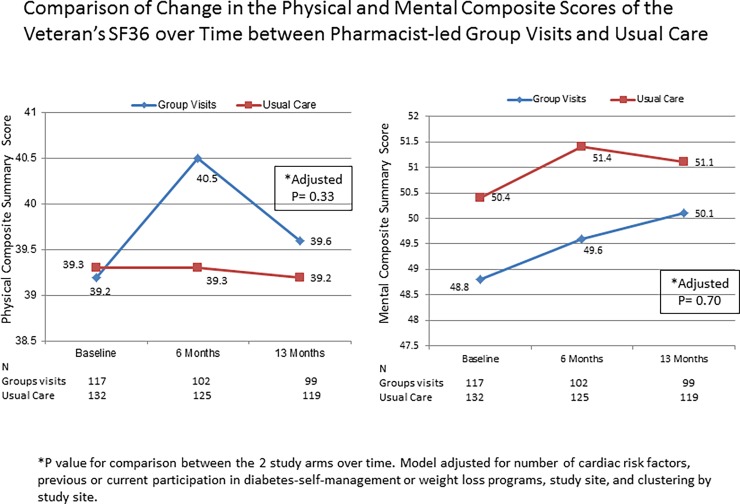
Comparison of change in the components of SF-36v over time between pharmacist-led group visits and standard care. **C**hange from baseline in health status was not significantly different between participants in the group visits or the standard care arms.

After 13 months of the trial, change from baseline in health status was not significantly different between participants in the group visit and the standard care arms (adjusted p = 0.33 for the physical composite score and p = 0.70 for the mental composite score; Fig 3).

### VHA health services utilization costs

**Fig 4:**
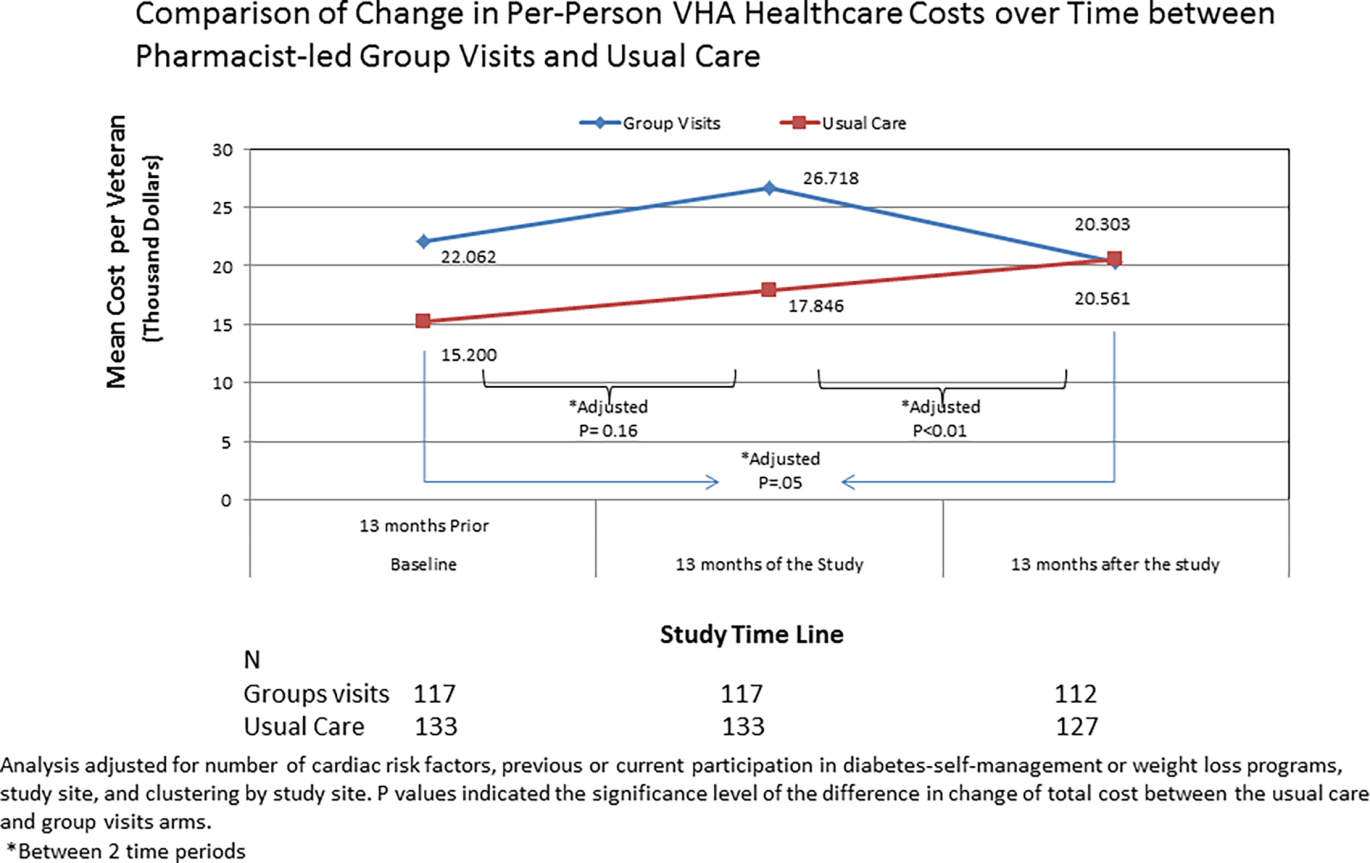
Change in healthcare costs during and after the study period between pharmacist-led group visits and standard care. When compared to baseline, the change in VHA healthcare costs was similar between the study arms during the 13 months of study interventions. Comparison of change in VHA healthcare costs 13 months after the intervention period versus 13 months of the intervention period, significant reductions in the overall VHA health service costs were found for the group visit arm whereas a steady rise in cost was found for the standard care arm.

The per-patient-cost of group visits during the13-month study period was $370 ±192. After 13 months of the trial, change from baseline in VHA health service’s costs per study participant was not significantly different between participants in the group visit and the standard care arms (+$4656 or +21.1% versus +$2645 or +17.4% per-person, group visit versus standard care, adjusted p = 0.16; Table 2). A breakdown of the costs showed that the increase in per-person outpatient expenditure from baseline was significantly lower in the group visit versus the standard care arm, both during the study intervention period (+$1629 versus +$1943; adjusted p = 0.04) and at 13-months after study interventions (-$795 versus +$501; adjusted p<0.01), respectively. In contrast, the per-person increase in medication expenditure from baseline was significantly higher in the group visit versus standard care arm during the study intervention (+$1213 versus +$318; adjusted p = 0.03), but became similar 13-months after the study intervention (-$331 versus +$655 per-person; adjusted p = 0.29)(Table 2, Fig 4).

**Table 2:** Change of VHA expenditure in healthcare costs of study patients.

	Group Visits (n = 117)	Standard Care (n = 133)	Adjusted P Value
Estimated Mean (SD) Total VHA Expenditure per Person, $			
Change in cost 13 months of Study period minus 13 months before the study	4656 ± 31881	2645 ± 25580	0.16
Change in cost 13 months after Study period minus 13 months of Study period	-1575 ± 30774	2360 ± 23708	<0.01
Change in cost 13 months after Study period minus 13 months before the study^a^	4220 ± 31091	5752 ± 29690	0.05
Estimated Mean (SD) VHA Outpatient Expenditure per Person, $			
Change in cost 13 months of Study period minus 13 months before the study	1629 ± 24188	1943 ± 9004	0.04
Change in cost 13 months after Study period minus 13 months of Study period	-795 ± 13224	501 ± 11170	<0.01
Change in cost 13 months after Study period minus 13 months before the study^a^	2838 ± 15008	2614 ± 12679	0.19
Estimated Mean (SD) VHA Inpatient Expenditure per Person, $			
Change in cost 13 months of Study period minus 13 months before the study	1520 ± 16529	-150 ± 17758	0.33
Change in cost 13 months after Study period minus 13 months of Study period	497 ± 20637	682 ± 14384	0.16
Change in cost 13 months after Study period minus 13 months before the study^a^	1491 ± 21758	1044 ± 15798	0.03
Estimated Mean (SD) VHA Pharmacy/Medication Expenditure per Person, $			
Change in cost 13 months of Study period minus 13 months before the study	1213 ± 8549	318 ± 2109	0.03
Change in cost 13 months after Study period minus 13 months of Study period	-331 ± 9622	655 ± 5463	0.29
Change in cost 13 months after Study period minus 13 months before the study^a^	808 ± 5950	1006 ± 5885	0.41
Estimated Mean (SD) VHA fee-basis (outsource referral/transfer) costs per Person, $			
Change in cost 13 months of Study period minus 13 months before the study	-75 ± 12580	535 ± 5264	0.30
Change in cost 13 months after Study period minus 13 months of Study period	-574 ± 9961	522 ± 7716	0.42
Change in cost 13 months after Study period minus 13 months before the study^a^	-917 ± 10892	1088 ± 6318	0.08

All tests conducted in log-cost. Analysis adjusted for number of cardiac risk factors, previous or current participation in diabetes-self-management or weight loss programs, study site, and clustering by study site. P values indicated the significance level of the difference in change of total cost between the usual care and group visits arms.

^a^n = 112 for group visit arm and n = 127 for Standard care arm

Exploratory analysis on the change in healthcare costs between the group visit arm versus the standard care arms showed significant reductions in favor of the group visit arm 13 months after the trial period versus the previous 13 months of the trial (-$1575 ±30774 or -5.9% versus +$2360 ±23708 or +13.2% per-person, adjusted p<0.01) and versus the baseline 13 months prior to the trial (+$4220 ±31091 versus $5752 ±29690, adjusted p = 0.05) (Table 2).

Sensitivity analysis using propensity-matching did not qualitatively alter the cost results.

### Safety

There were 24/117 (20.5%) emergency room visits and 26/117 (22.2%) hospitalizations for patients in the group visit arm and 24/133 (18.1%) emergency room visits (p = 0.62) and 21/133 (15.8%) hospitalizations (p = 0.19) in the standard care arm during the 13-month study period. There were 2/117 (1.7%) deaths in the group visit arm and 4/133 (3.0%) in the standard care arm (p = 0.50). Hyper or hypoglycemic events that required unscheduled visits to the health care provider occurred in 5/117 (4.3%) of patients in the group visit arm and in 7/133 (5.3%) of patients in the standard care arm (p = 0.71).

## Discussion

A 13-month pharmacist-led group medical visit intervention in Type-2 DM added to standard care was similar to standard care in achieving improvement from baseline in the overall cardiovascular risk composed of hemoglobin A1c, systolic blood pressure, and LDL cholesterol. There was no significant change from baseline in health-related quality-of-life between the two study arms. Despite this additional intervention, the increase in per-person outpatient expenditure from baseline was significantly lower in the group visit versus the standard care arm, both during the study intervention period and at 13-months after study interventions. The overall VHA healthcare costs/person were comparable between the study arms during the study period; then decreased by 6% for the group visit to levels lower than the baseline but increased by 13% for the standard care arm 13 months post-study.

Compared to our previous single-site studies of pharmacist-led group medical visits [18, 19], the current multi-site study failed to achieve a significant improvement over standard care in the overall cardiovascular risk. The reasons can be multi-factorial, part of which can be attributed to the smaller effect achieved in this multi-site effectiveness study (A1c reduction of -0.27% versus -0.9% in previous) [18, 19], the longer duration of the current study (with higher risk of regression to the mean) and the remarkable performance on A1c reduction by standard care (-0.14% versus 0.0% in previous) [18, 19]. These results are in contrast to the multi-site study by Edelman and colleagues with primary care physicians conducting group visits [13], which demonstrated a significant reduction in blood pressure over standard care, but from a mean baseline systolic pressure of 150’s mmHg. Our study population had lower mean systolic pressure (135’s mmHg) and LDL cholesterol levels (90’s mg/dL) at baseline and suggests that group visits may not be superior when baseline values are already at or near guideline recommended targets.

Diabetes is a chronic disease that requires frequent medical visits for control of glycemia and associated cardiovascular risk factors [27], which may explain the steady rise in healthcare costs in participants from both study arms during the study intervention period. However, pharmacist-led group visits had a lower outpatient expenditure compared to standard care, and overall lower healthcare costs after the study period to levels lower than baseline. We speculate that the multi-disciplinary care in the group visit program was potentially offsetting additional physician visits needed to achieve optimal chronic disease management. It has also been shown that the group setting helps in improving efficiency when it comes to education, lifestyle modification and discussion of care barriers as opposed to intervention to only one individual at a time [28, 29].

Group medical visits can also provide peer support of participants toward lifestyle changes, and a focused environment for diabetes care without the competing demands of non-diabetes related issues that often surface in routine primary care visits, and access to special expertise in diabetes education without going to another clinic appointment [30]. Of note, some of the healthcare cost savings in the group visit arm was offset by the rise in medication costs during the study period, which could be explained by an increased prescription activity and possibly patient adherence, a rise that stabilized after completion of the study interventions. Edelman and colleagues showed that a multi-disciplinary group medical visit model with physician providers resulted in cost-savings 2 years after the completion of the intervention [15]. Our study built on Edelman’s results to show that group visits can be led by clinical pharmacists without physician involvement, and health care cost reductions can be achieved after 13 months to below the baseline levels.

The findings also suggest that addition of pharmacist-led care was safe with similar hospitalizations and death compared to standard care, which can have significant implications. The capacity of the current health system to provide chronic disease care with an expanding diabetes epidemic is diminishing as the demand for health care providers increases [31, 32]. By allowing clinical pharmacists to practice to the “top of their license” in an integrated care delivery model such as the VHA’s [33], physicians can potentially focus on higher acuity, more complicated patients. We also showed how a weekly intensive phase achieved a rapid improvement in glycemic control, an effect that reached a plateau when switched to quarterly sessions. These findings suggest that the frequency of visits is proportional to the degree of disease control and would allow policy makers to make a balanced decision on the adequate follow-up frequency, likely accounting for the patient’s underlying risk of adverse events.

Our study has limitations. First, only ~40% of individuals contacted agreed to participate which suggests that group visits may not fit all patients with T2DM. Second, we included only patients who sought care in VHA hospitals, a cohort that is mostly male in a non-fee-for-service system. Third, we only included individuals who are suitable candidates for algorithm-based care for which these findings cannot be extrapolated to individuals with conditions within the exclusion criteria such as pregnancy or dialysis.

In conclusion, addition of pharmacist-led group medical visits in T2DM achieved similar improvements in cardiovascular risk factors than usual care while outpatient care costs decreased. Furthermore, thirteen months after the completion of the intervention, healthcare costs in group medical visits decreased to below baseline levels compared to the steady rise in cost in usual care. Future studies should be conducted in the non-VHA setting to assess the generalizability of the results.

## Supporting information

S1 Table CONSORT checklist. (PDF)

S1 Text Trial study protocol. (PDF)
