# Supplementary material for: Costs and effectiveness of pharmacist-led group medical visits for type-2 diabetes: A multi-center randomized controlled trial
Source: PLoS One. 2018 Apr 19;13(4):e0195898. doi: 10.1371/journal.pone.0195898 (PMC5908172; doi:10.1371/journal.pone.0195898)
Supplement: S1 Text — (PDF) [file pone.0195898.s002.pdf]

I. RESEARCH OBJECTIVES

Approximately 65% of diabetic patients die of cardiovascular disease,[1, 2] which is largely preventable by simultaneous control of multiple cardiovascular risk factors.[3-9] However, concomitant multi-factorial therapy using vigorous behavioral and pharmacologic interventions are labor intensive and costly,[1, 3, 10] and difficult to accomplish in traditional care settings.[10-12] Group medical visits have been proposed to be an efficient method to achieve cardiovascular risk reduction and guideline implementation in diabetes mellitus (DM), through efficient resource use, improvement of access to care, and promotion of behavioral change through group support.[13, 14] However, these treatment models require intensive physician involvement to show clinical efficacy, which may reduce the potential cost savings expected from this intervention.[15-17] We are proposing to integrate the concept of using clinical pharmacists in lieu of the physicians from the Chronic Care Model (CCM) into the group medical visits to manage diabetic patients and its associated cardiac risk factors.

We have performed preliminary studies that have shown 4 weekly sessions of such group-based, Multi-disciplinary Education in Diabetes and Intervention for Cardiac risk reduction (MEDIC) to be feasible and effective in improving hyperglycemia and other cardiac risk factors, and its effects persist for at least 3 months.[18] We also have pilot data to support the use of the MEDIC strategy on a quarterly basis for maintenance of cardiovascular risk control in patients that have been successfully treated for cardiac risk factors (ClinicalTrials.org protocol NCT00358033). However, we have not formally tested the effects of the weekly MEDIC interventions to treat DM and cardiac risk factors in a large randomized-controlled study, or to assess the effectiveness of its continuation after the 4 weekly sessions in maintaining cardiac risk control. This is important because we have found that cardiac risk control regresses towards usual care 1 year after its successful treatment in specialized clinics.[19] In addition, we have not performed health-related quality of life (hr-QOL) or cost identification analyses that would be needed for a widespread adoption of this “system” of care. Therefore, we propose to evaluate our MEDIC intervention vs. usual care for 13 months in a randomized controlled trial of type 2 DM patients who have a HbA1c >7.0%, and are non-adherent to American Diabetes Association (ADA) guideline recommendations in 1 or more cardiac risk factors, defined as being a current smoker, have a blood pressure >130/80 mm Hg, or an LDL cholesterol (LDL) >100 mg/dL.[8] The MEDIC intervention (experimental arm) will consist of Phase 1: 4 weekly group sessions of MEDIC followed by Phase 2: booster group MEDIC visits at 3 months intervals for 12 months. The control arm will continue on their usual care for the 13 months. We hypothesize that patients randomized to the MEDIC intervention will be more successful than usual care in reducing the risk of future coronary events as measured by the United Kingdom Prospective Diabetes Study (UKPDS) risk engine after 13 months into the study, at a minimal added VA institutional cost and with a significant increase in patient hr-QOL.[20] The UKPDS risk engine is used because it provides a single, comprehensive outcome measure that is applicable to DM patients with respect to cardiovascular risk, and its elements include age at diabetes diagnosis, sex, race, current tobacco smoking status, hemoglobin A1c (HbA1c), systolic blood pressure, and total cholesterol to HDL cholesterol ratio. Our specific aims are to compare the MEDIC intervention and the usual care arms at 13 months of the study:

1. The 1-year coronary event risk as measured by the UKPDS risk engine;
2. The hr-QOL as assessed by the VA version of the Medical Outcomes Study Short Form (SF-36V),[21, 22]
3. Total costs of care during the study, analyzed from the perspective of the VA Medical Centers.
4. To assess the long-term effects of the intervention, we will also collect patient outcomes and cost of care 2 years after the study conclusion.

II. BACKGROUND/CONTEXT

The achievement of guideline recommended goals in smoking, blood pressure, cholesterol and glycemia requires both a strong pharmacologic component targeting all 4 cardiac risk factors, as well as a behavior modification component.

1. Social Cognitive Theory: Behaviors to reduce cardiovascular risk and promote diabetes self-care such as diet, exercise, glucose self-monitoring, and smoking cessation are complex, and can be explained by constructs drawn from the Social Cognitive Theory (SCT).[23, 24] Bandura defines two cognitive processes in SCT: outcome expectation, defined as an individual's belief that a behavior would produce a certain result; and self-efficacy (efficacy expectation), defined as a person's belief that he/she has the ability to carry out that behavior. Therefore, the DM self-care behaviors can be explained by determinants at the personal level: skills, outcome expectations, expectancies, self-efficacy, reinforcements, reciprocal determinism and self-control; and at the environmental level: social or peer support, social norms and perception, social reinforcement, access to equipment and resources, health provider contact time, and health provider care quality.[25-30] Most effective behavior interventions are likely to result from a program that aims at the different levels of behavioral change.[31, 32]

In MEDIC, we will use education (cognitive restructuring) and counseling in a group setting to change outcome expectations and shape expectancies (values patients placed on the achievement of a behavior goal). Demonstration and coaching of their self-care skills may increase self-efficacy. Other principles utilized in our behavioral interventions include: goal setting, social and peer support, monitoring (self and external) to aid in stimulus control, social negotiation and reinforcement (self and vicarious). The interventions will be further discussed under METHODS, but the details of each session are described in the manual of procedures in the appendix.

2. Chronic Care Model (CCM): Delivery of such a multi-level and multi-targeted (HTN, DM, lipids and smoking) therapy for integral management of DM is labor intensive and likely costly, and requires a collaborative care approach with participation of a team of multi-disciplinary health-care professionals.[11, 12] The alignment of the different needs of an integral program to manage DM and the existing VA resources can be complex, but achievable using the service delivery constructs from the CCM.[11, 12] The CCM is a disease management approach comprised of 6 components: 1) care delivery system re-design (use of clinical pharmacists), 2) link of the patient to community resources (exposure of the patient to VA resources), 3) self-management support, 4) provider decision support, 5) use of electronic medical record system and 6) a system-wide commitment to quality.[11, 12] Some components of the CCM are already present in the VA system, such as electronic medical record system and organizational commitment to quality. But other components have not been successfully integrated or tested using

group intervention strategies in DM.[16] Our program intends to merge with the pre-existing infrastructure to complete the components of the CCM.

The MEDIC intervention combines the tenets of the SCT with those of the CCM. Through care delivery re-design (CCM), clinical pharmacists deliver behavioral interventions in a group setting, applying the constructs of the SCT to promote disease self-management (CCM). They collaborate with the physician and the nurse to become providers of DM care, and coordinate the multi-disciplinary personnel to deliver DM education. The advantage of the clinical pharmacists, especially in the VA system, is the ability to carry out the pharmacologic treatment plans formulated in conjunction with the primary care provider and according to the pre-established algorithms, and follow the patients for medication adherence; all of which ease the burden of the primary care providers (provider decision support). The encounter with the clinical pharmacists will enhance the provider contact time and provider care quality factors for DM care (environmental determinant of behavior); and potentially free-up time for primary care providers to enhance non DM-related care. The MEDIC intervention also links the patient to the VA resources (CCM), as the patients are acquainted to the dietician, the physical therapist and the nurse educator. They are also educated on the community resources (CCM). As such, the MEDIC intervention comprehensively addresses the behavioral and pharmacologic aspects of simultaneous management of DM, HTN, smoking and dyslipidemia within a systemic structure that promotes patient and system success in cardiovascular risk reduction in DM. In this way, we will fill the knowledge gap of how SCT and group intervention strategies can be successfully implemented through CCM to treat DM. The above concepts are illustrated in the figure below.

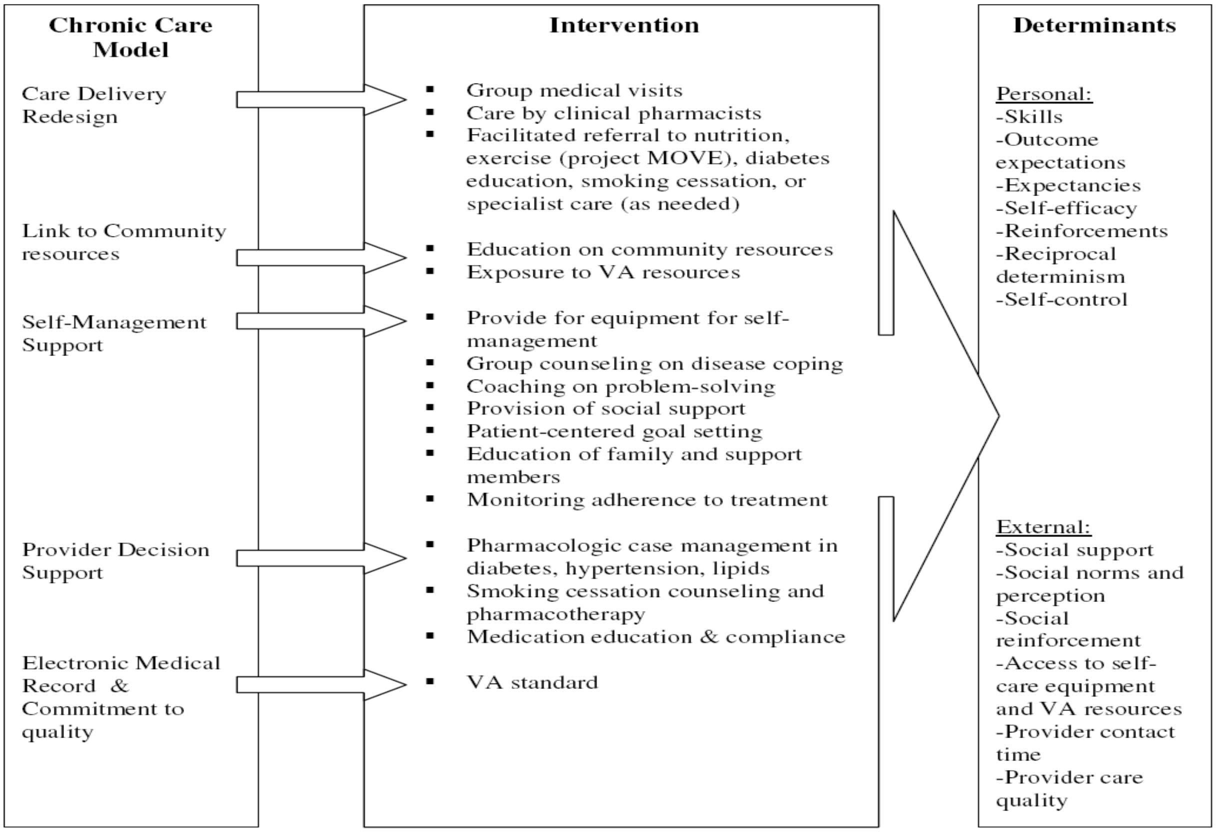

Figure 1. Theoretical Underpinning of MEDIC Intervention. Alignment of VA Resources with service delivery constructs of the CCM and intervention constructs drawn from the SCT. The VA resources are organized through MEDIC to provide multi-factorial interventions to effect behavioral changes and cardiovascular risk control in DM.

3. Group Intervention in Diabetes: Three basic group models were described in the literature to enhance DM care:

(a) Education-based clustered visits, which usually consists of group visits with a multidisciplinary team led by a nurse educator and may include a dietitian, a pharmacist, and a behavioral therapist.[15, 33-35] Education-based group visits without physician involvement and pharmacotherapy showed only modest improvements in glycemia with absolute HbA1C reductions between 0.32% to 0.43%.[33-35] When the interventions involve physicians to effect medication changes, HbA1c levels (baseline >8.5%) declined by 1.3% in the intervention arm versus 0.2% in the control arm (at 6 months).[15]

(b) Chronic-care clinics, which consist of individual (NOT group) appointments with the primary care physician, nurse, and clinical pharmacist, followed by group education and support by nurse.[16] After 2 years, this model may improve quality of life, DM knowledge and reduce specialty and emergency room visits, but did not improve HbA1C levels compared to controls (worsened from 7.4% to 7.9% in both groups).

(c) Group medical visits, where a physician and a DM educator provide group DM education every 3 months with individual physician follow-ups after the group sessions.[13, 17, 36] Pharmacologic interventions did not occur during the group sessions, but rather during the individual meetings with the physician after. After 2 years, A1C results of patients attending the group visits maintained their baseline levels, while those of control subjects deteriorated (baseline A1C for both groups 7.4 ± 1.4%; final A1C for intervention group 7.5 ± 1.4% vs. 8.3 ± 1.8% for control group).

The education-based clustered visits (a) and the group medical visits (c) were the only models that improved HbA1c levels over the control subjects, but both models required important physician time for pharmacotherapy and did not treat or improve on other associated factors such as smoking, blood pressure or dyslipidemia. Clinical pharmacists have shown effectiveness in managing DM, lipids and blood pressure in individual clinic visits,[37, 38] but their effectiveness in managing DM in a group setting is unknown, especially when the intention is to treat all cardiovascular risk factors in one single program. Our proposed model intends to answer: 1) whether targeted pharmacotherapy case management can be provided by clinical pharmacists following a pre-established treatment algorithm in a group setting, instead of physicians; 2) whether concomitant treatment of associated cardiac risk factors is efficacious in the group setting provided by non-physician staff; and 3) whether this intervention can be delivered by one provider to several patients at a time maximizing cost, instead of several healthcare providers intervening in the same group session, as described by previous models. If proven efficacious, this intervention model may represent potential cost-savings to the institution, while not compromising clinical efficacy.

In addition, our preliminary analysis suggests that there is a “break-in” period needed for patients and providers to feel comfortable in the group setting to receive or effect the changes, respectively. Therefore, we have built-in in our design the “intensive treatment” phase of weekly sessions for 4 weeks to account for this break-in period, followed by “booster” sessions every 3 months to maintain or even further the changes. Our literature review revealed that this type of design and differential intensity in the treatment to maximize cost and efficacy have not been previously described.[15],[16],[13, 17, 36]

Previous Work:

1. Individual Intervention - The Cardiovascular Risk Reduction Clinic (CRRC): The CRRC consisted of individual clinic sessions with clinical pharmacists, who are also certified DM educators to assist primary care providers control cardiovascular risk factors in patients with CAD or DM. The clinical pharmacists, who have prescriptive authority at the VA, provided behavioral counseling and medication titrations targeting HTN, DM, lipids and smoking using pre-designed treatment algorithms (appendix) in individual clinic sessions every six weeks. The CRRC improved glycemia, blood pressure, smoking and lipid control when compared to risk factor matched controls without CRRC intervention.[37, 39] However, regression of the cardiac risk control occurred 12 months after discharge from CRRC (figure 2).[19]

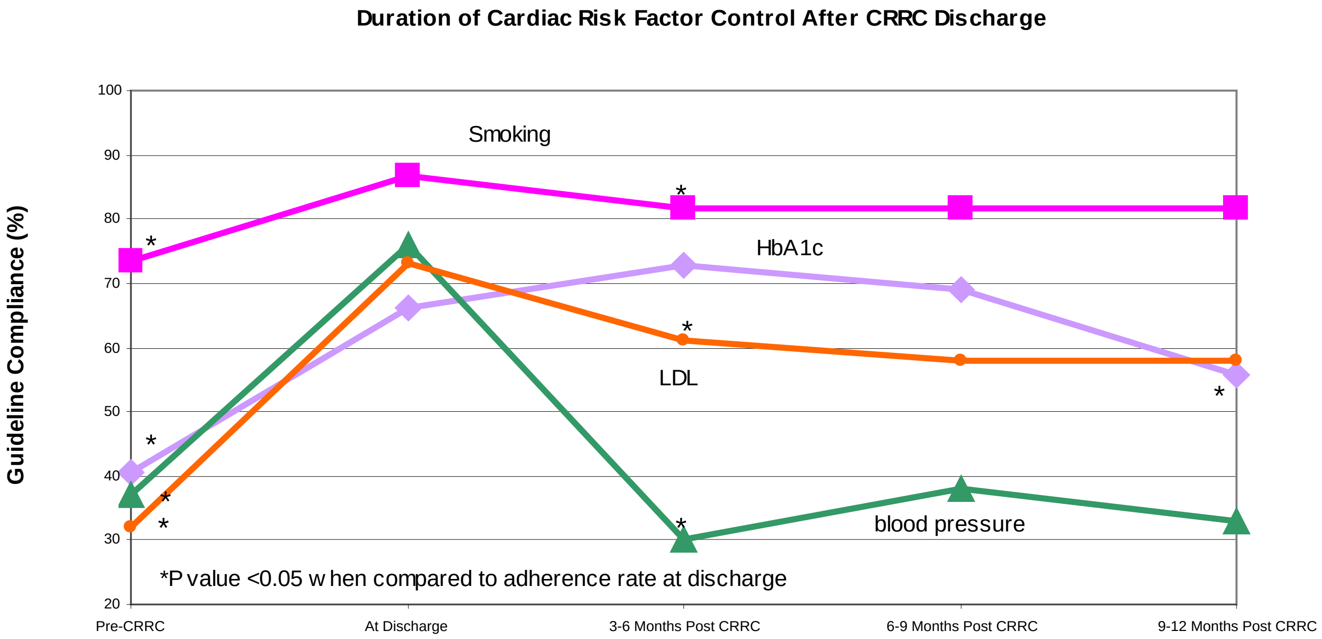

Figure 2. HBA1c, smoking, SBP and LDL achieve guideline compliance by the time of discharge from CRRC, but regression occurs at various rates during the first 12 months after discharge.

2. Group vs. Individual Intervention - Effectiveness of different strategies in maintaining target cardiovascular risk factors in patients discharged from CRRC: We prospectively pilot tested our MEDIC intervention strategy against individual CRRC treatment model given at 3 month intervals and against usual care, to compare their effectiveness in maintaining cardiac risk control for patients already at goal in their cardiac risk factors. Our preliminary data suggest that quarterly MEDIC interventions performed fairly well when compared to CRRC interventions in maintaining cardiac risk control, and both were superior to usual care.

	SBP at CRRC Discharge	SBP 12 Months After CRRC Discharge	HBA1c at CRRC Discharge	HBA1c 12 Months After CRRC Discharge	LDL at CRRC Discharge	LDL 12 Months After Discharge
CRRC n = 34	123.2± 10.8	125.9± 16.9	6.24± 0.69	6.77± 1.12	76.50± 17.82	81.20± 16.96
MEDIC n = 33	122.0± 8.9	128.3± 13.5	6.32± 0.61	7.19± 1.25	82.27± 18.56	86.13± 29.69
P = NS, CRRC vs. MEDIC and at discharge vs. 12 months after discharge. SBP = systolic blood pressure						

We calculated the ratio of the future coronary event risk at 12 months / baseline as measured by the UKPDS risk engine and found the MEDIC intervention to be superior (1.38 ± 0.76) to usual care (1.50 ± 0.61) in maintaining cardiac risk control.

3. Group Intervention – Weekly MEDIC - Pilot: We have preliminary results (both retrospective and prospective) to show that providing educational-behavioral interventions and medication titration in a group setting is feasible and effective in treatment naïve patients to improve cardiac risk factors after 4 weekly sessions.[18, 39]

**MEDIC Intervention**	SBP at Baseline	SBP After 3 months	HBA1c at Baseline	HBA1c After 3 months	LDL at Baseline	LDL After 3 months
Retrospective study n = 41	134 ± 19	130 ± 15	8.5 ± 2.2	7.0 ± 1.3*	100 ± 25	95 ± 45
Prospective study n = 24	132 ± 14	124 ± 18	8.1 ± 0.8	7.2 ± 0.8*	90 ± 30	85 ± 29
P = <0.05, measurements at baseline vs. after 3 months. SBP = systolic blood pressure.						

The ratio of the future coronary event risk at 3 months / baseline as measured by the UKPDS risk engine was 0.77± 0.41 in our retrospective study and 0.78 ± 0.42 in our prospective pilot study vs. 1.04 ± 0.38 for the control arm (in the prospective pilot study).

These pilot data and experiences suggest that although individual intervention models with the clinical pharmacist such as CRRC is effective, it is limited by patient's lack of knowledge of the disease and self-care, which makes initial CRRC sessions long and inefficient, and the need for multiple CRRC visits to achieve clinical efficacy. Additionally, referral to different health disciplines for education and counseling may impact on patient adherence due to the multiplicity of clinic visits. We have also found that regression of cardiac risk control occurs after discharge from CRRC, which makes the need for follow-up booster interventions necessary to maintain risk factor control. We have then created a group-based multi-disciplinary behavioral and pharmacologic intervention – MEDIC, from modification of the pre-existing DM Self Management Education (DSME) program integrated with the intervention components of our CRRC experience. Our data suggest that such a group intervention program is effective in improving cardiac risk and overcomes the barriers of patient's knowledge, multiple provider visits, and has an effect that lasts at least 3 months at a time. The multi-disciplinary approach utilized in this study will obviate the need for multiple clinic visits with different providers, as different providers are exposed to the patient in one program. In the current proposal, we intend to test the cardiac risk reduction effectiveness and sustainability of the MEDIC intervention in a larger study involving 2 VA sites, of longer duration, and using different clinical pharmacist providers. Although regression can still occur after the longer MEDIC intervention as part of the natural history of chronic illnesses, our study will provide the VA administrators the cost and efficacy data that are necessary for the long-term implementation of this intervention.

III. SIGNIFICANCE OF THE RESEARCH

Our program targets the VA priority of Disease Prevention and Guideline Implementation, specifically in the prevention of cardiovascular diseases in DM. We will investigate a novel strategy for the implementation of the CCM, through a group-based behavioral and pharmacologic intervention, a patient-oriented approach for DM guideline implementation. Diabetes mellitus affects 21 million people in the U.S. with an estimated health care cost of $92 billion.[1, 2] The prevalence of DM among veterans is approximately 12% and is associated with significant morbidity and mortality, especially cardiovascular complications.[40, 41] We have found that among diabetic veterans at the Providence VA, at least 21.8% are non-adherent to ADA Guidelines in multiple cardiac risk factors. Among patients with DM and concomitant coronary disease, 52% had an HbA1c >7%, 50% had a blood pressure >130/80 and 48% had an LDL >100 mg/dL.[42] Type 2 DM accounts for more than 95% of all diagnosed cases of DM, especially in the veteran population, and is the focus of our research.[2, 37]

Studies have consistently shown that control of cardiovascular risk factors such as smoking, hyperglycemia, HTN, and dyslipidemia reduces the occurrence of cardiovascular complications in DM.[3, 4, 6, 43] However, VA annual clinical performance report in DM care suggest that guideline recommended goals are not being achieved both locally and nationwide.[44] Obstacles at different levels of the DM care process have been identified in the literature. At the patient level, there is non-adherence to interventions, lack of the disease knowledge, and mistrust of the treatment.[45-47] At the provider level, the physician who prescribes the medication does not have sufficient time to provide education, frequent follow-up visits and behavioral counseling to ensure adherence of the medical and lifestyle regimens.[28-30] At the institutional level, the resources necessary to invest in staff training and the infrastructure necessary for the multi-factorial management of DM is major and not always available.[48]

Our program is important because it can be implemented using currently available non-physician staff with minimal additional training requirements, as each health professional is still performing their original function, but organized in a more efficient manner to deliver care. This approach will maximize available institutional resources and overcome barriers at different levels of the care process in DM, at minimal incremental cost.[13, 14] Group interventions have been tested in the past by Health Maintenance Organizations to reduce the cost of treatment and improve quality of care. Models such as the Kaiser's drop-in group medical appointment and cooperative health care clinic, Group Health's chronic care clinics and shared medical appointments in community group practices have all been explored outside the VA settings.[49-54] However, these treatment models require intensive physician involvement to show clinical efficacy in DM, which may reduce the potential cost savings.[15-17] Our model will use clinical pharmacists in lieu of the physicians and nurses for behavioral and pharmacotherapy in DM to achieve larger cost savings for the institution.

There are also VA initiatives for DM group intervention. Dr. David Edelman is currently conducting a VA HSR&D funded study "Can Group Visits Improve Outcomes of Veterans with Diabetes". While our models have in common the use of group intervention strategy to support primary care providers in the management of DM and cardiac risk factors, there are also important differences. These differences are: 1) in

the MEDIC proposal, clinical pharmacists lead the group intervention sessions and provide pharmacotherapy, while in Dr. Edelman's project, clinical pharmacists do not lead nor make independent pharmacotherapy decisions; 2) since patients already have their own primary care providers, there is no proposed additional physician involvement aside from consulting roles in the MEDIC proposal; while additional physicians play an essential role in leading the group intervention and pharmacotherapy in Dr. Edelman's project; 3) treatment intervals vary with time in the MEDIC proposal, as we are proposing a front-loaded treatment approach consisting of 4 weekly sessions followed by booster sessions every 3 months, to achieve clinical efficacy at a faster rate based on our preliminary findings; while Dr. Edelman's project proposes regular monthly visits to maintain the same intensity of the interventions throughout; 4) Our models also differ in the treatment of smoking, which is part of the MEDIC proposal, but not a component of Dr. Edelman's project. These differences can be potentially more cost-saving for the MEDIC intervention, without sacrificing efficacy. Since the organization of care for patients with DM is complex, it is unlikely that a single program could address all the needs and case scenarios in a "one size fits all" type. The need for one program vs. another, or both, will vary depending on the complexity of the co-morbidities at the patient level, the expertise of the providers and the availability of resources at the institutional level. Our program is likely to add to and complement the knowledge generated by Dr. Edelman's program, as there is a necessity for potentially less costly approaches for group interventions adapted to different needs.

Health-related quality of life (hr-QOL) is an important measure of an individual's sense of well-being, both physically and mentally, and is directly related to clinical outcomes.[55-58] Significant deficit in the hr-QOL is experienced by people with type 2 DM when compared to control population of similar age without the disease.[59] The proportion of decrease in hr-QOL increases in relation to the presence of complications of both microvascular and macrovascular origins. Despite its importance, few DM group intervention programs report on hr-QOL,[60] and virtually no data exist in the hr-QOL related to simultaneous treatment of HTN and dyslipidemia.[61] We will use this opportunity to collect data on how a group intervention strategy by clinical pharmacists may impact on the hr-QOL of patients with DM.

Cost identification is also imperative in assessing the potential for dissemination and implementation of any intervention program. Despite data from pooled analyses and randomized trials showing the cost-effectiveness of multiple cardiac risk control in DM,[3, 5, 62] there is intensive debate surrounding the best method to achieve those goals. Most arguments arise from inconsistent efficacy of different treatment programs and the lack of accurate analyses of program costs.[63] Less cost data exist for a group program such as ours, that contain medication titration without direct physician participation.[61, 64, 65] If successful, our program may decrease DM complications in the long-run, and may be cost-savings to the institution. More importantly, our model can be easily exported, as we use pre-existing human resources in clinical pharmacy, aid by educators in physical therapy, nursing and nutrition; all of which are available services in VA hospitals nationwide, with minimal additional training. It may also be sustained with minimal incremental cost. Moreover, the VA's commitment to quality of care is now being mimicked by major health care systems in the private sector.[66] Lessons learned in this program will have immediate applications in the private sector.

IV. METHODS

1. Overview: We propose to conduct a randomized-controlled study to test the effectiveness of a 13-month MEDIC intervention (experimental arm) vs. usual care (control arm) to reduce cardiac risk of patients with type 2 DM with a HbA1c >7.0%, and with either a blood pressure of >130/80, an LDL cholesterol >100 mg/dL, or being an active smoker. We will compare the hr-QOL, and average cost of care per person between the study groups. The study will last 13 months for all patients.

2. Population: Inclusion criteria: All diabetic veterans >18 years old with HbA1c >7.0% and at least one of the following: being a smoker (any cigarette smoking < 30 days), having an LDL >100 mg/dl or a blood pressure >130/80 mm Hg documented in at least two occasions within the last 6 months, able to participate and discuss their DM and cardiac risk control in a group setting and sign informed consent.

Exclusion criteria: Patients without eligible cardiac risk factors within the last 6 months; those who are unable to attend the group sessions; or disease conditions such as psychiatric instability (acutely suicidal, psychotic) or organic brain injury that preclude them from performing DM self-care. Patients with conditions that would preclude them from standard algorithm-based medication dose titrations such as those who are pregnant or with complex co-morbidities as defined by New York Heart Association Class 3 or 4 heart failure, liver cirrhosis, end-stage renal disease on dialysis and end-stage cancer will be excluded from the study. All women of childbearing age will have a pregnancy test before study enrollment. We will not exclude patients who were previously enrolled or are currently enrolled in DM education programs or project MOVE, as these patients would still benefit from the continuous medication titration and monitoring of treatment adherence by the MEDIC intervention. In addition, it would be important to know not only the efficacy, but also the effectiveness (in the “real life” setting) of the MEDIC intervention in the presence of co-interventions. Instead, we will account for their presence using stratified randomization, so equal proportion of patients participating in above-mentioned programs would be allocated to each study arm. We will exclude patients who are currently followed in a pharmacist led interventional clinic. However, if a patient is followed in a pharmacist led clinic and wishes to participate in the study, he/she may enroll if the patient and the provider agree that he/she would be withdrawn from the non-study pharmacist led clinic at the time of consent and randomization. However, since pharmacist-care are becoming popular and are considered part of "usual or standard care" in many VA facilities we will allow subjects to receive pharmacist-care during the study conduction period (from after the randomization to the end of the study) if so prescribed by their primary care providers, to reflect true "usual or standard care".

3. Recruitment: Patients will be identified by monthly HbA1c reports generated by CPRS (computerized patient record system) and recruited from 3 sites, the Providence (PVAMC), the West Haven VAMC (WHVA) and the VA Pacific Islands Health Care System (VAPIHCS. This research team has ample experience in patient recruitment using this strategy as this was used in previous MEDIC pilots. First, we will ask the IRB for a waiver of informed consent to screen for potential participants by CPRS chart review. We will then use CPRS query "create a custom search" command to create reports of patients with HbA1c levels >7% within the last 6 months. We will review their

medical records to ensure that they meet other study entry criteria. We will then send a "Dear Veteran" letter to eligible patients informing them about the study and that they will receive a phone call by our study staff within 1-2 weeks. Once contacted by phone, the veteran patient will be asked to come in for an initial study visit with the research assistant, where the study procedures will be explained to the patient and informed consent will take place. If the patient consents to the study, we will send their primary care physician a letter indicating their participation in the study. The site PI's will present informational sessions to all primary care and specialty providers during VA monthly meetings (Primary Care and Medicine) prior to the study kick off, so the patient's providers would have an a priori understanding of the study objectives. We will not retain any patient identifiers if the patient does not participate in the study. There will be no specific order of patient selection except for their study inclusion and exclusion criteria. Preliminary data showed that at least 1,310 patients with type 2 DM seen in the primary care clinics at the PVAMC in 2004 have 3 or more ADA non-adherent cardiac risk factors and met study inclusion criteria. Like PVAMC, the WHVA is part of the VA New England Healthcare System, has a large diabetic population (7,047 as of September '06), of which, 47% have a hemoglobin A1c >7.0%, >30% have an LDL >100 or no LDL at all, and >50% have a blood pressure > 130/85. They also have a similar history (4 years) of utilizing pharmacist-led group interventions in DM to manage the pharmacotherapy of these patients. Given the similarities of our systems and intervention models, and our long-history of collaboration in research trials, WHVA is an ideal second site for the study. PVAMC site will enroll 120 patients and WHVA site will enroll 100 patients for a total of 220 patients. Our multiple pilot studies in the past support the success of our research team in patient recruitment, retention and study execution.[37, 39] The pilot study program "Effectiveness of Different Strategies in Maintaining Target Goals of Cardiovascular Risk Factors" that we just completed, enrolled 72 patients in 12 months. Another pilot project "Weekly MEDIC Pilot)" started enrollment in July of 2005, and has recruited 70 patients in a 12-month period. Based on these experiences and the expectation that at least 25% of the qualified patients will consent to participate in the study, we expect our recruitment to be successful and complete by 24 months.

4. Randomization: Once enrolled, patients will be randomly assigned on a 1:1 ratio to either MEDIC (experimental arm) or usual care (control arm). It will be done by the call-in method to a central computer administered by the study coordinator, who will utilize "urn randomization" to stratify enrolled patients by the study site, the number of cardiac risk factors outside of the ADA recommendations (A1c levels, blood pressure levels, lipid levels and smoking) and previous or current participation to DSME, and project MOVE! to ensure balance between groups.[67] The "urn randomization" program allows researchers to randomize study subjects to 2 or 3 randomization groups while balancing on 2 to 20 variables (figure 3).

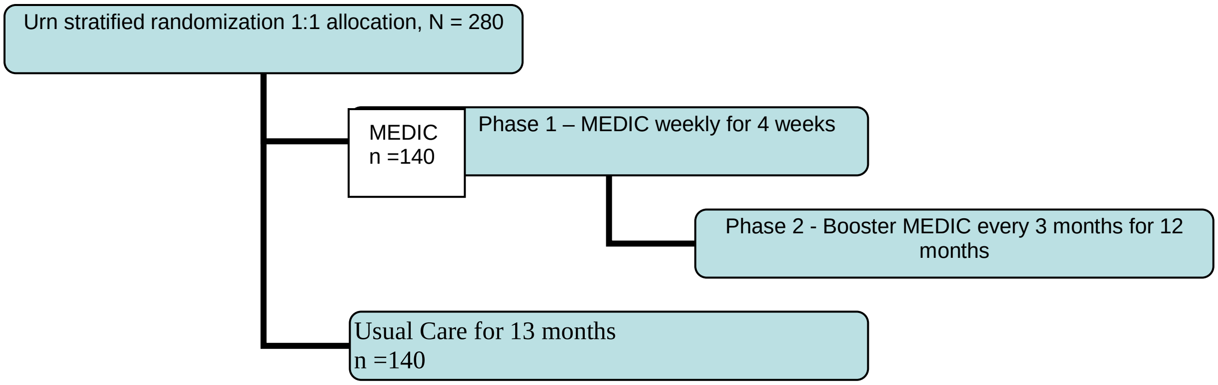

Figure 3. Study Flow Chart

5. Intervention: Patients in the experimental arm will receive two phases of MEDIC intervention (experimental portion), as an adjunct to the standard care of their primary care providers. Phase 1 has 4 weekly group sessions, and phase 2 consists of booster group visits every 3 months. The sessions are kept purposely informal and interactive to allow for open discussions about each individual's risk factor control, obstacles, and solutions. It will take place in a classroom setting and approximately 5 to 8 patients may participate in each group. Based on the SCT, the behavior of the members as a group led by the clinical pharmacist will influence on each individual's behavior, while each individual behavior will also influence the behavior of the group as a whole in a reciprocal fashion (reciprocal determinism).[68] Aside from counseling and coaching (with disease coping, problem solving, goal-setting), and teaching (skills of self-monitoring and self-control), the MEDIC team will also monitor the behaviors of the members of the group and provides reinforcement. The group setting will provide social and peer support, information and shaping of social norms and perceptions, reinforcement (both direct and vicarious), modeling and aid on the shaping of expectancies.[69-71] Since the family or support members are encouraged to be present in the group sessions, education is also provided to them to enhance social support for behavior change. The group visits are ideally suited to veteran patients since many of them share a common identity, lack social support, and are familiar with the group process through other VA treatment programs. In the group setting, the patients will be given resources (glucometer, sphygmomanometer, pedometer) and taught the skills to use them to perform self-care. They will be educated on the disease process and complications to shape outcome expectations. The supervised use of the different equipment of self care during the group sessions will enhance self-efficacy and facilitate the setting of new goals for disease self-care.

The success of the MEDIC intervention also depends on the coordination skills of the clinical pharmacist with collaboration from a multidisciplinary team of providers. The ability of different providers to work together as a team depends on the buy-in of the providers on the MEDIC intervention, effective coordination by the team leader and role definition of its members. We have the full-support of the respective Chiefs of Primary Care, nutritionist, physical therapy and nursing services from both study sites, who are also participants of the study. We have delineated the learning and intervention objectives for the sessions and the roles of different providers in the sessions in the revised study manual. We have also provided the team leader with suggestions on the

alternatives if some providers can not attend to the sessions (built-in redundancy).

5.1. Experimental Arm Phase 1– Weekly MEDIC: Sessions in phase 1 last 2 hours:

5.1.1. The first-half education (usually 40 – 60 min) consists of interactive lectures that contain an overview of DM, importance of diet, exercise, VA resources and DM complications and the treatment strategies to avoid complications. Although the lectures can all be provided by the clinical pharmacist, representatives of different services (dietician, physical therapist, and nurse) are invited to speak about their topics, so MEDIC patients can be exposed to the different resources within the VA and the mechanisms to access them. For example, Physical Therapy will demonstrate the use of exercise equipments and programs in the VA exercise room/gym during session 3. Specific referrals to the respective services or programs will be made on an as needed basis. The learning objectives are described for each session in the appended manual and follow the curriculum of the American Association of Diabetes Educators.[72]

5.1.2. The second-half behavioral and pharmacologic intervention (60 – 80 min) of DM, HTN, dyslipidemia, and tobacco utilizes behavior modification and medication case management approach in a group setting. It is provided by the clinical pharmacist and usually consists of the following segments:

- Review and reflection on the educational content of the lecture and group assessment of confidence and conviction in performing the target behavior
- Use of home monitoring equipment: patient demonstration of proficiency in the use of pedometers, glucometers and sphygomanometers, and documentation of results. Participants will not be provided any exercise equipment other than pedometers.
- Review of homework monitoring sheets and DM Report card: a discussion on the relationship between self-care behaviors and medications in the control of blood pressure, lipids, blood glucose, and tobacco cessation will be conducted in a question and answer format based on each individual's values in the report card. Behavioral goals will be set, and medication changes discussed. Family and social support are involved into the negotiation and implementation of goals. Any necessary facts will be cast in a problem-solving or behavioral context. Peer support and modeling, reinforcement, and coaching will be used to promote change.
- Problem Solving: The clinical pharmacist will help with disease coping, counseling, barrier identification and development of action plans for behavior change.[31, 32]
- Provide Homework: The goals of behavior change for the next meeting will be negotiated and written down here as homework.[73-75] Medication dose up-titrations will be written down in detail. Laboratory tests will be ordered when pertinent (about every other session). As described above, most of the interventions are based on the SCT. However, parts of the smoking cessation intervention are based on the transtheoretical model, as we provide patients handouts and homework based on the stage of change.[76] For current smokers or recent quitters (within 6 months) the homework format in the table below will be utilized:

For participants who are just being introduced of the topic of smoking cessation, have not set a quit date yet or the set quit date is greater than 6 months **(Pre-contemplation)**: the following phrase is written in their homework:<br>"Quit Smoking, So you can _____________ (personal goal such as: see your grand kids, save money, prevent erectile dysfunction, etc.)" The personal goal is derived from the interactive discussion. An example of incorporating a personal goal would be "Quit Smoking So You Can Walk without Your Legs Cramping"

For participants who have set a quit date that is within the next 6 months, but greater than 30 days **(Contemplation)**: Patients are instructed to limit their number of cigarettes. Generally, they are asked to decrease the number of cigarettes by 10 per day for the week if they smoke more than one pack per day. If they smoke less than a pack per day, they are asked to cut the amount of cigarettes in half.
For participants who have a quit date within the next 30 days **(Preparation)**:<br>a. Patients are provided with information about pharmacological options of helping them to quit (pre-established tobacco cessation treatment algorithm).<br>b. The patients are encouraged to decrease the number of cigarettes by 10 cigarettes per day if they smoke more than one pack per day. If they smoke less than a pack per day, they are asked to cut the amount of cigarettes in half.<br>c. They are asked to write down a quit date (this quit date will be reassessed in the following sessions).
For participants who have quit within the last 6 months (**Action phase**): The words “CONGRATULATIONS, You Should Be Proud of Your Success” are written very large on their homework sheet. Behavioral triggers that may sabotage their success and general recommendations to overcome urge to smoke are given in a handout format.

5.1.3. For participants in the VAPIHCS who resides in the Samoa site only: we will use telehealth technology (PicTel between Honolulu VAMC – American Samoa CBOC), in lieu of face to face meeting, to deliver the pharmacist-led group, with the help of an on-site nurse.

5.2. Experimental Arm Phase 2– Quarterly MEDIC: The structure of sessions in phase 2 will be similar to the weekly MEDIC described for phase 1, but without the first-half lecture component. It will last for 90 min instead of the previous 120 min per session and will occur every 3 months. The format of the behavioral modification sessions will be similar to the one described above. The goals consist of monitoring of the previously set goals in diet, exercise, blood glucose and blood pressure self-monitoring, and smoking cessation (when applicable). Barriers will be identified and solutions will be proposed. Reinforcement of desired behavior will be given. Medication titrations will occur for HTN, DM, lipids, or smoking cessation according to individual needs, the readings from the report card and laboratory data (appended manual).
5.3. Control Arm – Usual Care: The standard of care to patients with type 2 DM is provided by primary care providers through individual contact. There are about ~75 primary care providers (30 in PVAMC and 45 in WHVA) organized in “teams or firms (4 in PVAMC and 2 in WHVA)”. Each primary care provider has a panel size of ~1,200 patients; of which10-12% have DM. The primary care providers have access to on-line clinical tools and support resources such as drug formulary, electronic medical record system with clinical reminders (e.g. annual LDL cholesterol check for high risk patients, annual HbA1c check for diabetic patients, etc.), computer based reference material (e.g. Up-to-Date^{R}, Micromedex^{R}, MD Consult^{R}, PubMed^{R}), and referral services: cardiology, endocrinology, nutrition, physical therapy, pharmacy, DSME, MOVE!, ophthalmology, podiatry, social worker, and psychiatry. The duration of the primary care visits are scheduled at the discretion of the provider, which may vary from 20 to 60 minutes (average of 60 minutes) for a new patient and 30 minutes for follow-up patients. The frequency of the visits varies from weeks to months. In general, the average interval between primary care provider visits for patients with DM is 4 months. Subjects in the control arm will be offered an opportunity to participate in the MEDIC intervention after the study ends.
5.4. Subject Retention: Subjects will be reimbursed $100 for time and energy spent in filling out the questionnaires. Patients in both arms will receive monthly phone calls from

our research assistants to keep them engaged in the study. Aside from greetings, the phone calls will inquire about the use of non-VA services (inpatient and outpatient).

6. Outcomes: Our outcome from **specific aim 1** will be the 1-year coronary event risk as measured by the UKPDS risk engine. The UKPDS risk engine consists of:[20]

$$R_T(t) = 1 - \exp\{-qd^T (1-d^t)/(1-d)\},$$

where "$R_T(t)$" is the future coronary event risk over "t" years, in a patient with "T" years of diagnosed DM, in the absence of death from causes other than coronary heart disease. "d" is a constant representing the effect parameter for the duration of diagnosed DM (1.078 per year of diagnosed DM) and:

$$q = q_0\beta_1^{AGE-55} \beta_2^{SEX} \beta_3^{AC} \beta_4^{SMOK} \beta_5^{A1C-6.72} \beta_6^{(SBP-135.7)/10} \beta_7^{\ln(LR)-1.59},$$

where "AGE" = Age in years at diagnosis of DM; "SEX" = 1 for female, 0 for male; "AC" = 1 for Afro-Caribbean, 0 for Caucasian or Asian-Indian; "SMOK" = 1 for a current smoker of tobacco in any form at diagnosis of DM, 0 otherwise; "A1c" = hemoglobin A1c (%); "SBP" = Systolic blood pressure (mmHg); and "LR" = Total cholesterol/HDL cholesterol ratio.

Calculating the risk of coronary heart disease at 1 year (t=1) to simplify the equation results in:

$$R_T(1) = -qd^T$$
$$R_T(1) = -(q_0\beta_1^{AGE-55} \beta_2^{SEX} \beta_3^{AC} \beta_4^{SMOK} \beta_5^{A1C-6.72} \beta_6^{(SBP-135.7)/10} \beta_7^{\ln(LR)-1.59}) * d^T$$

We understand that this risk engine would not provide us with an accurate absolute risk of a future coronary event in the subgroup of patients with previously diagnosed coronary disease, but it will still provide us with a useful parameter of comparison among those patients.

Our outcome from **specific aim 2** will be the change from the baseline in the hr-QOL as assessed by SF-36V,[21, 22] at 13 months of study enrollment. The Veterans version of the Medical Outcomes Study 36-Item Short Form Survey (SF-36V) is a multi-purpose health status survey that addresses hr-QOL from physical and mental health perspectives.[21, 77] It yields 8 individual scales related to an overall assessment of the hr-QOL: 1) physical functioning, 2) role limitations due to physical problems, 3) bodily pain, 4) general health perceptions, 5) energy or vitality, 6) social functioning, 7) role limitations due to emotional problems, and 8) mental health;[22],[78] The eight scales of the SF-36V have Cronbach's alphas ranging from 0.69 to 0.89, with excellent discriminant validity of the individual scales and component summaries. More importantly, it has been found to be very responsive to the development of diabetic complications,[79] and is especially advantageous to compare outcomes in different diseases.[56] The SF-36v may be scored in several ways: as physical component summary (PCS) and a mental component summary (MCS), or as eight individual constructs.[21] Each subscale is graded from 0 to 100 points, with a score of 0 representing low functioning and 100 representing high functioning.[21] The PCS and MCS constructs are calculated using published algorithms, which weigh the composite scores and transform the scores following a t-distribution to a mean of 50 and standard deviation of 10 for the normal population. We prefer to use SF-36V in our study over DM-specific instruments, because the former is more robust, allows for inter-disease comparisons (our study

population has concomitant HTN and dyslipidemia),[80] has long-term outcome data which allow us to make future projections, and has been repeatedly validated for research use in the veteran population.[81, 82] We consider it to be especially suited for our study population, who has a much older age (average = 70.1 years) than the general DM population and a higher prevalence of pre-existing DM complications. For example, the prevalence of coronary disease was 24.7% among diabetic patients based on our 2004 preliminary data (n= 6,010), and the prevalence of renal dysfunction was also high with an average creatinine clearance of 72 ml/min (estimated based on an average weight of 92.5 kg).

Our outcome from **specific aim 3** will be the average cost of the intervention per patient from the VA (payer) perspective during the 13 months of study enrollment. Since our pilot studies have shown the efficacy of our MEDIC intervention vs. usual care in lowering the UKPDS risk engine score from baseline, the economic analysis for this project will be a cost-identification analysis, to be performed contingent on a confirmation of efficacy in this larger study. In the cost-identification analysis, we will compare the average cost of the intervention per patient for each arm of the study, analyzed from the perspective of the VA Medical Centers. Evaluating the costs from the VA perspective will allow VA managers to better understand how a change in therapy will affect short term (13 months) budgets. This is especially important with respect to VA policy, as VA managers are most concerned about the immediate budget effects. This analysis will require ongoing data collection and analysis during the study. Costs will include direct treatment costs, including inpatient, outpatient and pharmacy. The cost of the intervention and standard care will be estimated and compared using the micro-costing method.[83] Clinic visit and hospitalization data will be gathered from a variety of sources, including the Decision Support System (DSS) National Data Extracts and the VA National Patient Care Database SAS extract files ("OPC" and "PTF"). Because VA patients may obtain health care outside the VA system during the study, and the cost of such care must be taken into account, we will use the Fee Basis file to capture all care provided outside the VA and paid for by VA. Our statistical programmer (JL) has permission to access these datasets at the Austin Automation Center and will link this data using real SSN's. Since patients may obtain health care outside the VA that is not paid by the VA, and this may be relevant for the generalization of findings outside of the VA settings (but not within the VA), research assistants will perform monthly phone call interviews to capture this data in case report forms (hospitalizations, nursing homes, ER visits, medical procedures, and office visits).[84] Costs of medical services not paid by the VA will be assigned using a combination of data from Medicare reimbursement rates for specific types of services[85] and estimation conducted using the Medical Expenditure Panel Survey adjusted for medical price inflation as necessary.[86,87]

7. Study Visits and Data Collection: All patients will be seen in the enrollment visit by a research assistant to sign consent, orientation, and obtain socio-demographic information, SF-36V, weight, waist circumference, DM history, smoking history and baseline laboratory testing (HbA1c, lipid profile, blood count, hepatic and renal function and urine analysis). Baseline blood pressure measures will be obtained during that visit using a calibrated, automated sphygmomanometer. It will be measured in both arms first and the arm with the highest blood pressure will be used for the subsequent determinations. It will be measured 3 times, each 2 minutes apart, with final blood

pressure calculated as the mean of the second and third measured values. Research assistants must demonstrate proficiency in blood pressure determinations using the automated cuff, patient interviewing and data collection techniques to the site PI's.

Data about the participation of patients in DSME, project MOVE! or alike health promotion programs out of the VA after enrollment into our study will be tracked through chart review (within the VA) and phone interview (out of the VA).

Process outcomes: Baseline measures of self-efficacy in DM self-care and 7-day self-report of DM self-care behavior (diet, exercise, blood-glucose testing, and smoking) will be obtained using the "Perceived Competence in Diabetes Scale" and "The Summary of Diabetes Self-Care Activities Measure", respectively.[88-90]

The "Perceived Competence in Diabetes Scale" is a short, 4-item questionnaire to assess the subjects' "self-efficacy" or confidence in their ability to manage their DM care,[88, 91] a key construct of the SCT linking Self-management support from CCM.[92] It is well-validated in patients with DM with significant correlations (-0.35) with A1c levels and an excellent internal consistency (Cronbach alpha of 0.84-0.87).[88] For each of the items, the scale ranged from 1 (not at all true) to 7 (very true) and the score is calculated by averaging the responses on the four items. Higher scores indicate higher confidence in DM care.

The revised Summary of Diabetes Self-Care Activities Measure is also a brief (11 items), self-report questionnaire of DM self-management behaviors.[90] It has been validated in several studies for different DM self-care behaviors, for example the validity coefficient (r) for diet is at 0.25-0.50 range and for exercise is at 0.2-0.58 range.[27, 93, 94] The reliability among the different subscales (diet, exercise, blood-glucose testing, foot care and smoking) ranges from 0.30 for foot care to 0.75 for blood glucose testing.[93, 94] The score is based on the mean number of days per week (scale = 0-7) the subject performed the specific self-care behavior. For the smoking behavior (item 11), it is scored as 0 (= no), and 1 (= yes), and then the number of cigarettes per day was asked.

Since depression and anxiety symptoms may exert an important effect on risk factor control and adherence to treatment, we will also evaluate for baseline depression using the Centers for Epidemiologic Studies Depression Scale (CES-D) as it may directly influence patient's compliance and adherence to self-care.[95] The CES-D consists of a 20-item questionnaire validated against the longer questionnaires by Beck and Zung.[96, 97] Scores range from 0 to 60, with scores 16 or greater widely used as a threshold for clinical depression. Internal consistency for this measure in several different populations is relatively high, 0.85-0.90.[98]

Data Collection	Baseline	6 Months	13 Months	Data Source
Blood Pressure, HBA1c, Fasting lipid profile, Smoking, weight, waist circumference	X	X	X	Patient
Perceived Competence in Diabetes Scale and The Summary of Diabetes Self-Care Activities Measure	X	X	X	Patient
CES-D	X			Patient
SF-36V	X	X	X	Patient

Charlson Comorbidity Index	X			Chart
Outpatient and Inpatient VA service utilization	Ongoing			Dataset
Co-intervention Programs in the VA	Ongoing			Chart
Utilization of Non-VA paid medical services	Ongoing			Monthly phone interviews

Providence Site only:

Since patients with diabetes often have cardiac disease that is unrecognized, we would like to perform an ECHOCARDIOGRAM (Ultrasound of the heart) to characterize the heart function of our enrolled patients at the Providence VA Medical Center. This test is of minimal to no additional risk to study participants. Each subject will be asked to sign an additional consent addendum if they choose to participate in this one time procedure. The echocardiogram can be scheduled at any time during the 13 month study period that is convenient for both the subject and the echo technician.

After the initial visit, all participants will have another study visit at 6 months and an end of study visit at 13 months. To assess the long-term effects of the study intervention, we will also extract with chart review the study data described above 2 years after study conclusion. Since patients will not be contacted during this additional data extraction phase, data that requires additional patient contact such as questionnaires, interviews or surveys will not be collected. The outcomes in specific aim 1 (measures in HbA1c, lipid profile, and blood pressure, and smoking history for the UKPDS risk engine calculation) and specific aim 2 (SF-36V) in the follow-up visits will be obtained by a second research assistant blinded to treatment arm allocations. Self-reported changes in smoking cessation will be verified using saliva cotinine tests. The samples will initially be saved in a freezer and then sent to Salimetrics, Inc. for analysis.

Medical co-morbidities based on the Charlson Co-morbidity index, amount of co-payment for care, degree of service connection, and access to care (hospital-based clinics or community-based clinics), will be collected through chart abstraction by our research assistants to minimize participant burden. The Charlson Co-morbidity index is a weighted index that takes into account the number and the seriousness of comorbid disease. [99] The index is well-validated for its ability to predict risk of death from co-morbidities at 1-year and 10-year follow-up periods.

Data collection for cost will be gathered from a variety of VA sources. Datasets such as the DSS, the Fee basis files, the OPC and the PTF will be accessed from the Austin Automation Center at the patient level through real SSN's (JL). All inpatient and outpatient experiences within the VA will be captured through the OPC and PTF files. Patient prescriptions will be found in the DSS Pharmacy Extract. Facility and administrative costs for MEDIC will be adjudicated by the average overhead per clinical encounter of the clinic site at which the intervention is given, e.g. if MEDIC uses facilities and administrative support from Firm A in WHVA, it will assume the overhead per clinical encounter in Firm A. This overhead data is constantly being generated for each VA facility and is located in the DSS.[100] The DSS overhead data cannot account for differences in the duration of the clinical encounters and will assume that they all have equal weight. The cost of staff time will be derived from each provider's annual wage. The staff time for every MEDIC provider and educators (including the research

assistant's time when working on the MEDIC activities) will be tracked quarterly using activity logs and will include the time spent during the sessions, and the time spent in the preparation and follow-up, including case discussion sessions and chart documentation of the clinical encounters.[83]

All data will be collected using a study code that does not identify the patient. The key matching the patient with the code will be saved into a password-protected file, separate from the rest of the data files, inside secured VA servers in a locked room maintained by IRM in the respective sites; and only the PI's and the study coordinator has access to. All the collected study data will be saved in a file on the VA network (vhaprofpc14\research\MEDIC). Paper data will be stored in a locked cabinet within a locked room accessible only by the research team. In the event that theft, loss of other unauthorized access of sensitive data or storage devices and non-compliance with security controls occur, study staff has been instructed to follow the Providence VA Medical Center's standard operating procedure on incidence reporting. Records will be maintained in accordance with the Department of Veterans Affairs Record Control Schedule 10-1. When study personnel are no longer part of the research team they will be restricted from access to the research study data files and electronic access to personally identifiable information will be revoked.

8. Analytic Plan: The main analyses presented in this proposal are to examine the 13-month effect of MEDIC intervention vs. usual care on outcomes of our 3 study aims. Initially, descriptive statistics such as the minimum, maximum, range, median and mean for each variable are used to summarize the variable as well as detect outliers, data entry mistakes, and missing values. Exploratory graphical techniques such as Boxplots, Histograms, Quantile-Quantile plots, and Stem and Leaf plots will be used to further examine these data. Analyses will follow the intention to treat principle. Analyses will be performed using SAS 9.13 and Stata 9® . The following specific aims will be modeled using multivariate logistic regression analysis described below.

8.1. Specific Aim 1: We will compare the 1-year coronary event risk as measured by the UKPDS risk engine between the study arms. This aim is the most important and clinically relevant as it requires successful treatment and follow-up, and therefore, used as the basis for our sample size calculations. T-test comparing the event risk of patients in both arms will be conducted. It is possible that non-parametric (Wilcoxon) methods will be required if the distribution of the differences appears not to be normally distributed. We will use General Estimating Equation (GEE) from Liang and Zeger,[101, 102] to model the treatment effect on the outcome controlling for variables which are imbalanced at baseline: demographics (age, race, gender), co-morbidities (Charlson Comorbidity index), participation to co-intervention programs (e.g. DSME and MOVE!), UKPDS score, SF-36v score, weight and waist circumference, co-payment, service connection, access (hospital-based or community-based clinics), and a fixed effect for the study sites.[103]

8.2. Specific Aim 2: Similar analyses described in specific aim 1 will be used to compare the change from baseline to13 months in the SF-36V scores between patients in both study arms. Both the change in summary scores and subscales (8 domains for SF-36V) will be compared using T-test (Wilcoxon if needed). GEE will be used to model the treatment effect on the change of SF-36v scores controlling for variables which are imbalanced at baseline mentioned above: demographics, Charlson Comorbidity index,

and weight and waist circumference, participation to co-intervention programs, UKPDS score, SF-36v score, co-payment, service connection, hospital-based or community-based clinics, and a fixed effect for the study sites.

8.3 Specific Aim 3: Since our pilot studies have shown the efficacy of our MEDIC intervention vs. usual care in improving the probability of coronary-event-free survival as measured by the UKPDS risk engine, the economic analysis for this project will be a cost-identification analysis, to be performed contingent on the confirmation of efficacy in this larger study. In the cost-identification analysis, the average cost per person of the intervention in MEDIC will be compared to the average cost per person of the intervention for usual care using T test (Wilcoxon, if needed) analyzed from the perspective of the VA Medical Centers.[83] Mean cost per patient (in dollar amounts) will be estimated using short-run costing, therefore, cost projections over time such as capital depreciation or long-term savings will not be considered. Since the distribution of the cost data is often skewed, logarithmic transformation of the data will be performed when necessary. Additionally, since our aim is to perform a cost identification analysis and not a cost-effectiveness analysis (which would need estimated total costs and weighted cost estimations for censored data), the cost of patients who dropped out would not be taken into account when estimating the average costs per person of the intervention. Confidence intervals around these mean estimates will also be calculated to indicate precision of the estimate. Multivariate modeling will be used to control for variables which are imbalanced at baseline: demographics, Charlson Comorbidity index, participation to co-intervention programs, UKPDS score, SF-36v score, weight and waist circumference, co-payment, service connection, and access. We will focus on the accounting cost as it is the most relevant to the implementation decision. The cost components derived from the interventions have been described above and include: staff time, overhead, laboratory testing, and institutional resource utilization including, all inpatient and outpatient services, and medications and supplies. Additional analyses will be performed incorporating the cost data on medical care incurred in Non-VA settings and not paid by the VA (captured by monthly phone interviews) to assess for generalization of MEDIC in non-VA settings. Costs incurred for research purposes such as participant reimbursement, statistician or research coordination will not be included. The tracking of cost with the addition of Hawaii sites will rely heavily on Fee-Basis files since the local VA relies heavily on outsourcing to the local military and community hospitals.

We will test the sensitivity of our results to a number of assumptions, including changes in costs due to changes in the study site, medical price inflation, study personnel and the severity of underlying illnesses (UKPDS, SF-36 score).

8.4. Additional Analyses: We will also compare the proportion of patients who achieve ADA recommended goals in blood pressure, HbA1c, LDL cholesterol and smoking cessation between the study arms. Sensitivity analyses of the effectiveness of the intervention will be explored among different subgroups, which will include, but not limited to: the study site, number of cardiac risk factors, depression, self-efficacy (by Perceived Competence Scale), and participation to co-intervention programs.

8.4.1. Mediating factor analyses: In order to understand the mechanisms by which the MEDIC intervention improves UKPDS risk engine scores, we will explore the following potential mediators from The Summary of Diabetes Self-Care Activities

Measure: change from baseline in diet, exercise, blood-glucose testing, and smoking; and the change in the degree of self-efficacy (by Perceived Competence Scale). In order to prove mediation, four conditions must be met: (1) correlation between the change in treatment variable (MEDIC intervention or not) with the change in mediator (change in DM self-care activities or self-efficacy); (2) variation in the mediator must be correlated with variation in the outcomes (UKPDS risk engine); (3) controlling for the mediator substantially reduces or eliminates the relationship between the treatment and outcomes; and (4) the treatment variable must temporally precede the mediator, which is inherent in our study because enrolled patients will be treatment naive.[106] We will compare multivariate regression model specifications that serially exclude and include the potential mediators and the treatment variable. We use the multivariate delta method to estimate the standard error of mediated effects.[108, 109]

8.4.2. Moderating factor analyses: We will also explore the following potential moderators of treatment response: level of depression by CES-D, participation to co-intervention programs, and self-efficacy (Perceived Competence Scale). GEE analyses described for specific aim 1 above will be performed in a staged approach. We will use the 1-year coronary event risk as measured by the UKPDS risk engine as the dependent variable. The baseline regression models with its corresponding covariates and the treatment variable will be entered on the first step of the analyses. Potential moderators will then be entered into separate prediction equations (one equation for each potential moderator) on the second step of the analyses. Lastly, the interaction term of treatment condition multiplied by the moderating variable will be entered as a predictor variable. Prediction equations that include the moderating variables will be compared to equations that do not include these variables, to assess the amount of variance (R2 and adjusted R2) in treatment outcome accounted for by these variables, compared with the amount of variance accounted for by treatment group allocation.

8.4.3. We will compare patient’s cardiovascular and diabetes outcomes between the study arms, 2 years after the study end. Similarly, we will also compare patient’s health care utilization costs 2 years after the study end.

8.5. Missing data: Since these are primary data collected as part of a clinical trial, we do not anticipate significant missing data. Overall (with the exception of the cost data), if the number of observations with missing values is small (<5%), we will conduct analyses by removing those observations with missing values. If the number of observations with missing values is many, we will resort to methods that impute values assumed to be missing at random.[110] However, if the data are not randomly missing, various methods will be evaluated. If significant differences are found in the attrition rates across study arms, both ‘intention to treat’ and ‘as treated’ analyses will be preformed to determine the extent to which the missing data may be biasing the results. For the ‘intention to treat’ analysis, we will generate actual raw data values suitable for filling in gaps in an existing database. Typically, five to ten databases are created in this fashion. We will then analyze these data matrices using an appropriate statistical analytic method, treating these databases as if they were based on complete case data. The results for these analyses are then combined into a single summary finding. SAS Proc MI and MIANALYZE readily impute maximum likelihood estimates from incomplete data.[111, 112]

8.6. Power calculations: Our 12-month pilot, (project 2 under "Previous work"), assessed the efficacy of quarterly sessions of MEDIC vs. individual interventions vs. controls in maintaining target goals for 12 months in patients already treated for their cardiac risk factors. Using the UKPDS risk engine on patients assigned to the quarterly group sessions for 12 months (n = 66), we estimated the mean and SD of the 1-year coronary risk after the intervention. Since we do not have the data for the date of the onset of diabetes, we calculated the risk assuming the age of diagnosis of diabetes is the same age of the patient at the time of the study enrollment. We found that the mean coronary event risk estimated by the UKPDS risk engine in these patients to be 0.04 (SD 0.1). Assuming equal number of patients in each study arm (by design) and equal standard deviations (confirmed by our pilot studies), the table below describes the difference of the means that our proposed sample can detect at different attrition rates.

**2-sided alpha of 0.05, 90% Power**	**Attrition (%)**	**N (each arm)**	**Difference of Means**
	**0**	**140**	**.0391**
	**10**	**126**	**.04403**
	**20**	**112**	**.0429**
	**30**	**98**	**.0460**

Our increased study sample will also provide us with robustness to violations of model assumptions, protection against multiple comparison error rates, and increased efficiency to detect weaker relationships between variables.

9. Minority and Gender Assurances: There are no exclusion criteria based on race or gender. Given that both PVAMC and WHVA provide services for over 80,000 veterans in the entire Rhode Island, Connecticut and South Eastern Massachusetts catchments' areas, we are confident that our study will be able to recruit adequate numbers of minority populations representative of the current veteran population with DM. We do not plan to oversample based on gender or race as our own data have shown that the gender and self-reported race distribution of our diabetic patient population is representative of the overall veteran population with cardiac conditions and risk factors.[116] It is 98.4% male, 5.4% African American, 0.3% American Indian or Alaska native, 77.5% for Caucasian, and 5.1% for Hispanic (11.7%=unknown).

10. Limitations: First, a concern with an intent-to-treat analysis of long-term outcomes is that differential loss-to-follow-up in the treatment and control arms may exist. We have previously addressed how missing data will be handled and have also accounted for as much as 25% attrition rate in our sample size calculations. Second, it is possible that secular trends such as an increase in the organizational pressure to use of guideline recommendations by providers or increased in the availability for co-interventions threaten our experimental design and cause underestimation of our treatment effects. We are using stratified randomization to balance previous or current co-interventions and will track this carefully during the enrollment period. Adjusted analyses for imbalanced in exposure to co-interventions at baseline will be performed

when necessary and stratified analyses by co-interventions will also be explored as detailed in our analysis plan. In addition, it is likely that the effects of secular trends should be randomly distributed in a randomized controlled study, such that co-interventions should not have a differential effect. Third, It is possible that our study duration is not long enough for our patients with DM to develop sufficient complications for us detect a significant change in the hr-QOL using SF-36V. However, given the older age and co-morbidities of our patients, we consider our chance of detecting a change in hr-QOL is fairly good. It will also provide us with an unique opportunity to evaluate the hr-QOL effects as a result of our proposed interventions in lifestyle modifications and intense medication regimen. Fourth, there is always a risk of not achieving the targets for patient recruitment. Fortunately, the project team is very experienced, and has had excellent track records in patient recruitment and retention in previous studies of similar characteristics. The addition of WHVA as a second site will provide us with additional protection to recruitment goals.
11. Ethical aspects of the proposed research: Dr. Wu and his research team are all up to date with the **HIPAA and Good Clinical Practice Trainings.** IRB approval will be sought prior to chart screening of potential participants. Identifiers will be destroyed if potential candidates do no enroll. We will obtain patient's written consent for their participation. All VA sensitive information will be stored in secured VA servers. Once the data are collected, patients will be coded with their identifiable information erased. Only the site PI's and the study coordinator will have the original code assignment, which will be safeguarded in a password-protected file in the VA servers. Any paper data will be filed in locked cabinets inside locked rooms that only the PI's have the key access and will be destroyed after 5 years of study completion. No patient identifiable information will be released or published without written permission unless required to do so by law. We consider the risk of our project to be mild to moderate, mainly social, when breech of subject confidentiality occurs due to unforeseen circumstances; and psychological when patients may become uncomfortable when discussing about their illnesses or lifestyles in a group setting. There may not be direct benefits to patients participating in this study, although there is a chance that our intervention may lead to improved cardiac risk management and, therefore, a reduction in the risk of heart attack and stroke.

In order to collect study data through chart review 2 years after the study period, we will ask IRB for waiver of HIPAA and informed consent. We will not contact the study subjects during this chart review phase of the study.

V. DISSEMINATION / IMPLEMENTATION PLAN

The dissemination and implementation of our proposed interventions will be performed via direct and indirect dissemination (meetings and publications) methods.
1. Presentation of our findings at National VA meetings: We anticipate presenting at the 4th year of the grant at the VA HSR&D meetings in February, in D.C., and National non-VA scientific meetings: American Heart Association Health Services and Outcomes Research meeting in May, in D.C. or the ADA annual scientific sessions in June.
2. Publication in peer-reviewed journals: Once funded, our trial will be registered in ClinicalTrials.gov. Authorship requirements in each manuscript will be published as

outlined by the recommendations of the International Committee of Medical Journal Editors.[117] The order of authors in the publications will follow the criteria listed by the committee, and will be a joint decision of the coauthors. We anticipate the writing of several articles during the 4th year of the grant, which will include but not limited to:

a) "Efficacy of a pharmacist-based DM group intervention program for cardiac risk reduction" will analyze the efficacy of the group intervention in diabetic cardiac risk reduction and its comparison with usual care.

b) "Group Intervention in DM Collaborative Care: Intervention and Implementation" will describe the theory and experiences in the implementation of MEDIC in primary care. This will serve as the procedural manual for disseminable implementation to other VA hospitals, VISN and nationwide (draft manual in appendix).

c) "Diabetes group intervention and effects in health-related quality-of-life to achieve adherence in multi-factorial cardiac risk reduction", intends to analyze the effect of MEDIC in hr-QOL, by the end of 13 months.

d) "Cost implications in the implementation of DM group intervention program for guideline implementation", will provide a provider perspective cost evaluation of the MEDIC program in comparison to usual care.

e) "Mediating factor analyses of the MEDIC intervention", intends to analyze the factors that mediate the change in UKPDS scores in MEDIC intervention. A separate manuscript will address the moderators of the MEDIC intervention.

Notification of paper approvals will be submitted to HSR&D prior to publications.

3. Direct Dissemination: our results will be presented at multiple levels of VHA leadership for immediate implementation:

a) Medical Center Level: The results of the study will be presented to the Hospital Director, the Chief of Medicine, Chief of Primary Care, Clinical Pharmacy, Nutrition, and nursing of the respective study sites at month #39 of the study. This will be facilitated by Drs. Sharma (Chief of Acute Care and Specialty Service Line Director, VISN-1), O'Toole (Chief of Primary Care at PVAMC), Federman (Chief of Primary Care at WHVA), Foody (Cardiology, WHVA) and Friedmann (Director of Target Research Enhancement Program (TREP) at PVAMC). Dr. Sharma is a cardiologist with expertise in the conduction of multi-center cardiovascular clinical trials. Through his position as the Director of Acute Care and Specialty Service Line, he is extremely involved in the guideline implementation practices through out VISN-1 and will provide a strong clinical expertise and administrative leadership in the dissemination of results at the local VA and within the VISN. If proven effective, we will recommend automatic referral to MEDIC of patients with DM and cardiovascular risk factors not at target goals. We will track blood pressure, LDL, smoking and HbA1c outcomes data at 6 month intervals through the automated reporting of the VA electronic medical record system (VISTA). This automated data reporting system is already up and running for Dr. Wu and his team in PVAMC to generate preliminary data for the design of our study.

b) VISN Level: If effective, we will present MEDIC to the department heads of Medicine at the Specialty and Acute Care Service Line monthly meetings chaired by Dr. Sharma and the Primary Care Service Line meetings at month #41 of the study. Dr. Wu will lead this dissemination process with the support of the service-line Chiefs. We will also present our findings to VISN heads of Pharmacy, Nursing, and Nutrition. This report will include recommendations on the training for execution of MEDIC and on the

cost-reducing features. PVAMC will provide training for clinical pharmacists to run the group sessions. Dr. Tracey Taveira, who had been conducting MEDIC sessions for the last 4 years and who has trained Pharmacy residents and students at the PVAMC to conduct MEDIC, will conduct targeted 3-day hands-on orientation sessions with emphasis on group dynamics and behavioral and pharmacologic interventions in a group setting. A draft of the study manual (appendix) has also been completed to support these implementation efforts. These sessions will be co-instructed by the PI and Dr. Gopalakrishnan, our endocrinologist. Traveling costs for these seminars can be solicited from the education funds within each medical center. Dr. Taveira's previous trainees have taken positions as primary care clinical pharmacists at North Hampton VA and are ready to implement MEDIC. The publication of the study manual will facilitate this dissemination process. The study investigators will visit the major VISN hospitals to aid on the set-up of MEDIC on solicitation. Outcomes of the VISN implementation will be tracked through automated reporting, simulating the system already in PVAMC.

c) VHA nationwide: The nationwide implementation of MEDIC will be performed through a multi-site randomized-controlled study using the Cooperative Studies Program (CSP) or the Service Directed Research (SDR) mechanisms. Dr. Mark Bauer has extensive experience using CSP dissemination by being the study chair of the CSP #430 "Reducing the Efficacy-Effectiveness Gap for Bipolar Disorder", which implemented group and individual interventions for patients with bipolar disorders using a collaborative care approach.[118, 119],[120] Dr. Charles Eaton is the PI of an ongoing NIH grant to intervene at the primary care provider level (30 primary care practices) for implementation of cholesterol adherence guidelines for cardiovascular disease prevention in Southeastern New England and has ample experience helping the team coordinating efforts for study dissemination at the private sector. Dr. John Piette, a nationally renowned expert in DM disease management programs, a researcher at the Center for Practice Management and Outcomes Research and member of the VA DM QUERI, will aid Dr. Wu the dissemination at the VA through the QUERI mechanism. Dr. Kim Gans, an NIH-funded researcher in clinical trials of lifestyle modification for cardiac risk reduction and former member of the Pawtucket Heart Health Program, who already assisted Dr. Wu in the refinement of MEDIC interventions and the manual development, will also help in the broad implementation of MEDIC. Data in this study and experience in the VISN implementation of our model will serve as the preliminary data for our CSP or SDR application. We will start this process in the last 6 months of the grant.

VI. PROJECT MANAGEMENT PLAN

The project will last 4 years. The study sites will be the PVAMC and WHVA. It will be divided in 3 parts: randomized controlled trial, data analysis, and dissemination of results. Although all investigators are involved in all the aspects of the study, specific committees are formed to take advantage of each person's expertise. Dr. Wu is ultimately responsible for the coordination and execution of the entire research program. The PI and his research team have been collecting data on the proposed strategies in smaller scale through several feasibility trials at the PVAMC as described above. WHVA is also an experienced site with all the components of the research and clinical team in place, and has been performing group interventions in DM for 4 years. The required

infrastructure and manpower organization are already in place and the teams have the capability to start the intervention upon funding decision.

Monthly investigator meetings, in form of conference calls (VANTs line) of one hour duration, will take place to discuss the progress of the project and future objectives to be achieved. Face to face meetings will take place quarterly, both in PVAMC and WHVA, in an alternate fashion. Informal weekly local team meetings will take place in each of the respective sites to troubleshoot both clinical and administrative issues. E-mail or telephone communications will take place throughout the study between all the team members. The Gantt chart outlines the key study tasks and activities.

1. Randomized-controlled trial: The randomized controlled trial will take place in the first 36 months, and includes patient screening, recruitment, and conduction of the trial. Patient screening and recruitment will be performed by our research assistants and the randomization by our research coordinator. Drs. Federman, O'Toole, Friedmann and Eaton will form **the study executive committee** to oversee the conduction of the study and assist Dr. Wu in the coordination of the administrative and logistic aspects of trial (resources, space and problem solving). Dr. Federman is the Chief of Primary Care at WHVA. Dr. O'Toole is the Chief of Primary Care at PVAMC. Both Drs. Federman and O'Toole are experienced researcher administrators, and will help Dr. Wu in overcoming administrative and logistic hurdles in the implementation of this project. Dr. Friedmann is a health services researcher, strongly supported by the VA and the NIH in the field of health-care organization, behavioral change and the psychosocial aspects of chronic diseases. He and Dr. Eaton will advice Dr. Wu in the conduction of this multi-site trial.

The **clinical team** is formed by the multi-disciplinary staff of clinical pharmacy, nutrition, and nursing from both WHVA and PVAMC, led by Dr. Tracey Taveira at PVAMC and Dr. Sean Jeffery at WHVA, and will provide direct patient care in MEDIC. Drs. Wu, Foody, Holt and Gopalakrishnan will be assisting Drs. Taveira and Jeffery monitoring the clinical aspects of the trial locally, with discussion of the difficult cases and management scenarios as well as the re-design of the treatment algorithms when necessary. Drs. Gopalakrishnan and Holt are endocrinologists for PVAMC and WHVA, respectively. Drs. Wu and Foody are cardiologists at the PVAMC and WHVA sites. Selected case discussions with the study physicians will occur weekly. We anticipate finalizing the recruitment by 24 months and the trial phase by 36 months. Treatment fidelity will be assessed according to a pre-established checklist (included in the appended manual) for the MEDIC sessions and scored (% completion of the items) by our experienced study coordinator, Lynn Marquis (4 CSP's and numerous industry-based grants, and IRB member). Lynn will work closely with the study executive committee to ensure that study procedures are followed by each site. For each of the MEDIC clinical pharmacists (WHVA and PVAMC), fidelity will be assessed in 4 weekly sessions and 4 quarterly sessions. The sessions will be considered compliant when the average % completion of the items is at least 80%.

2. Data Management and Analysis: Data abstraction will follow the progress of the trial and involves 2 components: abstraction from Austin by our programmer (JL), and direct patient measurement and interview, phone call interview and chart abstraction from research assistants. Our study coordinator will monitor and ensure the overall adequacy of patient data collection and record keeping. Data cleaning and analysis will begin at month 31 of the study and will continue throughout. Drs. Wu, Sinnott, Piette

and Intrator will be in charge of the **data management and analysis committee**. A statistical programmer (JL) led by Dr. Intrator will be specifically in charge of the statistical analysis. Dr. Intrator is the biostatistician at the TREP at PVAMC, has 37 peer reviewed publications in access to health care, hospital resource utilization in the nursing home population, and an expert in the use of Monte-Carlo simulation for economic analyses. Drs. Sinnot and Piette will provide additional expertise in the abstraction of the Austin datasets related to cost and hr-QOL variables, and economic analysis of the data. Dr. Sinnott is a health economist at HERC, and is very experienced in the use of different VA databases for cost-identification analyses.

Data files will be organized with the variables coded. Descriptive statistics will be performed on the variables obtained and a dictionary of the complete set of variables will be assembled. We expect to obtain our initial results during months 37-39.

3. Dissemination and Implementation: Drs. Wu, Sharma, O'Toole, Foody, Piette, Bauer, Gans and Federman will form **the dissemination and implementation committee** to disseminate our work at different fronts. The preparation of the efficacy of the group intervention article and the costing analysis will be the priorities to facilitate implementation of our intervention. Local implementation will occur at 38-39 months led by Drs. O'Toole and Federman. VISN-1 dissemination will occur during the same time and implementation will begin at months 40-42 led by Drs. Wu, Sharma and Foody. Months 43-45 will allow us to troubleshoot and overcome unforeseen obstacles during VISN wide implementation. The encountered problems will be recorded as well as collection of preliminary outcome data. The organization for the application to the CSP and/or SDR programs in for VA nationwide implementation will start at months 43 led by Drs. Wu and Foody and supported by Drs. Bauer, Gans and Piette. Annual reports to HSR&D Central office documenting the progress of the project will be written. We will conclude the study by the submission of the Project Final Report.

LITERATURE CITATIONS

1. Centers for Disease Control. The Burden of Chronic Diseases and Their Risk Factors: National and State Perspectives 2004. Atlanta: National Center for Chronic Disease Prevention and Health Promotion; 2004.
2. American Heart Association. Heart and stroke statistics -- 2005 update. Dallas, Texas: American Heart Association; 2005.
3. Gaede P, Vedel P, Larsen N, Jensen GV, Parving H-H, Pedersen O. Multifactorial Intervention and Cardiovascular Disease in patients with type 2 diabetes. New Engl J Med 2003;348(5):383-93.
4. The Diabetes Control and Complications Trial/Epidemiology of Diabetes Interventions and Complications (DCCT/EDIC) Study Research Group. Intensive diabetes treatment and cardiovascular disease in patients with type 1 diabetes. New Engl J Med 2005;353(25):2643-53.
5. The CDC Diabetes Cost-effectiveness Group. Cost-effectiveness of intensive glycemic control, intensified hypertension control, and serum cholesterol level reduction for type 2 diabetes. JAMA 2002;287(19):2542-51.
6. UK Prospective Diabetes Study (UKPDS) Group. Efficacy of atenolol and captopril in reducing risk of macrovascular and microvascular complications in type 2 diabetes: UKPDS 39. BMJ 1998;317(7160):713-20.
7. National Cholesterol Education Program. Third Report of the Expert Panel on Detection, Evaluation, and Treatment of High Blood Cholesterol in Adults (Adult Treatment Panel III). NHLBI, 2004. (Accessed July 6th, 2005, at http://hin.nhlbi.nih.gov/cvd_frameset.htm.)
8. American Diabetes A. Standards of Medical Care in Diabetes-2006. Diabetes Care 2006;29(suppl_1):S4-42.
9. Chobanian AV, Bakris GL, Black HR, Cushman WC, Green LA, al. e. Seventh report of the Joint National Committee on Prevention, Detection, Evaluation, and Treatment of High Blood Pressure. Hypertension 2003;42:1206-52.
10. Rosenzweig J, Weinger K, Poirier-Solomon L, Rushton M. Use of a disease severity index for evaluation of healthcare costs and management of comorbidities of patients with diabetes mellitus. Am J Manag Care 2002;8:950-8.
11. Wagner E, Austin B, Davis C, Hindmarsh M, Schaefer J, Bonomi A. Improving chronic illness care: translating evidence into action. Health Aff (Millwood) 2001;20:64-78.
12. Bodenheimer T, Wagner E, Grumbach K. Improving primary care for patients with chronic illness. JAMA 2002;288:1775-9.
13. Weinger K. Group medical appointments in diabetes care: is there a future. Dia Spectr 2003;16(2):104-7.
14. Jack L Jr. Diabetes Self-Management Education Research. An international review of intervention methods, theories, community partnerships and outcomes.An international review of intervention methods, theories, community partnerships and outcomes. Dis Manage Health Outcomes 2003;11(7):415-28.
15. Sadur CN, Moline N, Costa M, et al. Diabetes management in a health maintenance organization. Efficacy of care management using cluster visits. Diabetes Care 1999;22(12):2011-7.

16. Wagner EH, Grothaus LC, Sandhu N, et al. Chronic care clinics for diabetes in primary care: a system-wide randomized trial. Diabetes Care 2001;25(4):695-700.
17. Trento M, Passera P, Tomalino M, et al. Group Visits Improve Metabolic Control in Type 2 Diabetes: A 2-year follow-up. Diabetes Care 2001;24(6):995-1000.
18. Martin OJ, Wu WC, Taveira TH, Eaton CB, Sharma SC. Effectiveness of pharmacist-coordinated care to reduce cardiovascular risk factors in patients with diabetes mellitus. Diabetes Educator 2007;33(1):118-2.
19. Wu W, Martin O, Taveira T, Schleinitz M, Mor V, SC SS. Durability of guideline adherence in coronary disease and diabetic patients after discharge from a cardiovascular risk reduction clinic. Circulation 2004;109:e285,P41(abstract).
20. Stevens RJ, Kothari V, Adler AI. The UKPDS risk engine: a model for the risk of coronary heart disease in Type II diabetes (UKPDS 56). Clinical Science 2001;101:671-9.
21. Ware JE Jr, Snow KK, Kosinski M, B. G. SF-36 health survey: manual and interpretation guide. Boston: The Health Institute; 1993.
22. Jones D, Kazis L, Lee A, et al. Health status assessments using the Veterans SF-12 and SF-36: methods for evaluating outcomes in the Veterans Health Administration. J Ambul Care Manage 2001;24(3):68-86.
23. Bandura A. Social foundations of thought and action: a social cognitive theory. Englewood Cliffs, New Jersey: Prentice-Hall; 1986.
24. McKenzie JF, Smeltzer JL. Planning, implementing, and evaluating health promotion programs. A primer. Needham Heights, MA: Allyn and Balcon; 2001.
25. Baranowski T, Cullen KW, Nicklas T, Thompson D, Baranowski J. Are current health behavioral change models helpful in guiding prevention of weight gain efforts? Obesity Research 2003;11:23S-43S.
26. Chapman-Novakofski K, Karduck J. Improvement in knowledge, social cognitive theory variables, and movement through stages of change after a community-based diabetes education program. J Am Diet Assoc 2005;105:1613-6.
27. Nelson KM, McFarland L, Reiber G. Factors influencing disease self-management among veterans with diabetes and poor glycemic control. J Gen Intern Med 2007;22:442-7.
28. Mosca L, Linfante A, Benjamin E, et al. National Study of Physician Awareness and Adherence to Cardiovascular Disease Prevention Guidelines. Circulation 2005;111:499-510.
29. Hobbs FR, Erhardt L. Acceptance of guideline recommendations and perceived implementation of coronary heart disease prevention among primary care physicians in five European countries: the Reassessing European Attitudes about Cardiovascular Treatment (REACT) survey. Family Practice 2002;19:596-604.
30. Grant RW, Devita NG, Singer DE, Meigs JB. Polypharmacy and medication adherence in patients with type 2 diabetes. Diabetes Care 2003;26(5):1408-12.
31. National Cancer Institute. Theory at a glance. A guide for health promotion practice. In: US Department of Health and Human Services, ed.: National Institutes of Health; 2005.
32. Glanz K, Rimer B, Lewis F. Theory, research, and practice in health behavior and health education. 3rd ed. San Francisco: John Wiley & Sons, Inc.; 2002.
33. Sarkadi A, Rosenqvist U. Experience-based group education in type 2 diabetes: a randomized controlled trial. Patient Education and Counseling 2003;53:291-8.

34. Ellis SE, Speroff T, Dittus RS, Brown A, Pichert JW, Elasy TA. Diabetes patient education: a meta-analysis and meta-regression. Patient Education and Counseling 2004;52:97-105.
35. Gary TL, Genkinger JM, Guallar E, Peyrot M, Brancati FL. Meta-analysis of randomized educational and behavioral interventions in type 2 diabetes. The Diabetes Educator 2003;29(3):489-501.
36. Noffsinger FB, Atkins TN. Assessing a group medical appointment program: a case study at Sutter Medical Foundation. Group Pract J 2001;50(4):42-8.
37. Taveira TH, Wu WC, Martin OJ, Schleinitz MD, Friedmann P, Sharma SC. Pharmacist-led cardiac risk reduction model. Prev Cardiol 2006;9(4):202-8.
38. Lee JK, Grace KA, Taylor AJ. Effect of a Pharmacy Care Program on Medication Adherence and Persistence, Blood Pressure, and Low-Density Lipoprotein Cholesterol: A Randomized Controlled Trial. JAMA 2006:296.21.joc60162.
39. Misra B, Gopalakrishnan G, Martin O, et al. Cardiovascular risk reduction programs for patients with diabetes. Circulation 2005;111:e209, P80(abstract).
40. Weinstock RS, Hawley G, Repke D, Feuerstein BL, Sawin CT, Pogach LM. Pharmacy Costs and Glycemic Control in the Department of Veterans Affairs. Diabetes Care 2004;27(suppl_2):B74-81.
41. Pogach LM, Hawley G, Weinstock R, et al. Diabetes prevalence and hospital and pharmacy use in the Veterans Health Administration (1994). Use of an ambulatory care pharmacy-derived database. Diabetes Care 1998;21(3):368-73.
42. Patel P, Wu W, Cheng J, Martin O, Taveira T, Sharma S. Pattern of care of outpatients with coronary artery disease in a VA hospital. (abstract). Circulation 2004;109:e231,P184.
43. Group HPSC. MRC/BHF Heart Protection Study of cholesterol lowering with simvastatin in 20,536 high-risk individuals: a randomised placebo-controlled trial. Lancet 2002;360(9326):7-22.
44. FY 04 - Abbreviated Executive Summary - the "Xmas tree". VA New England Healthcare System, 2005. (Accessed July 9th, 2005, 2005, at http://vaww.visn1.med.va.gov/Templates/Inner.aspx?pid=735.)
45. Balkrishnan R, Rajagopalan R, Camacho F, al. e. Predictors of medication adherence and associated health care costs in an older population with type 2 diabetes mellitus: a longitudinal study. Clin Ther 2003;25:2958-71.
46. Wroe A. Intentional and unintentional non-adherence: a study of decision making. J Beh Med 2002;25:355-72.
47. Hernandez-Ronquillo L, Tellez-Zenteno JF, Garduno-Espinosa J, Goanzalez-Acevez E. Factors associated with therapy noncompliance in type-2 diabetes patients. Salud Publica Mex 2003;45(3):191-7.
48. Jackson G, Yano E, Edelman D, Krein S, Ibrahim M, et al. Veterans Affairs Primary Care organizational characteristics associated with better diabetes control. Am J Manag Care 2005;11:225-37.
49. Scott JC, Conner DA, Venohr I, et al. Effectiveness of a group outpatient visit model for chronically ill older health maintenance organization members: a 2-year randomized trial of the cooperative health care clinic. J Am Geriatri Soc 2004;52:1463-70.

50. Scott JC, Robertson BJ. Kaiser Colorado's Cooperative Health Care Clinic: a group approach to patient care. Manag Care Q 1996;4(3):41-5.
51. Beck AT, Scott JC, Williams P, et al. A randomized trial of group outpatient visits for chronically ill older HMO members: the Cooperative Health Care Clinic. J Am Geriatri Soc 1997;45(5):543-9.
52. Coleman EA, Eilertsen TB, Kramer AM, Magid DJ, Beck AT, Conner DA. Reducing emergency visits in older adults with chronic illness: a randomized, controlled trial of gropu visits. Eff Clin Pract 2001;4:49-57.
53. Bronson DL, Maxwell RA. Shared medical appointments: increasing patient access without increasing physician hours. Cleveland J Med 2004;71(5):369-77.
54. Noffsinger FB. Physicians shared medical appointments: a revolutionary access solution. Group Pract J 2002;51(1):1-9.
55. Wilson I, Cleary P. Linking clinical variables with health-related quality of life. A conceptual model of patient outcomes. JAMA 1995;273:59-65.
56. Ware JE Jr. Conceptualization and measurement of health-related quality of life: comments on an evolving field. Arch Phys Med Rehabil 2003;84:s43-s51.
57. Sen S, Fawson P, Cherrington G, et al. Patient satisfaction measurement in the disease management industry. Disease Management 2005;8(5):288-300.
58. Alazri M, Neal R. The association between satisfaction with services provided in primary care and outcomes in type 2 diabetes mellitus. Diabetes Medicine 2003;20:486-90.
59. Holmes J, McGill S, Kind P, Bottomley J, Gillam S, Murphy M. Health-related quality of life in type 2 diabetes (TARDIS-2). Value Health 2000;3 Suppl 1:47-51.
60. Tahbaz F KI, Calvert D. An audit of diabetes control, dietary management and quality of life in adults
with type 1 diabetes mellitus, and a comparison with nondiabetic subjects. J Hum Nutr Diet 2006;19:3-11.
61. Norris SL, Glasgow RE, Engelgau MM, O'Connor PJ, D. M. Chronic disease management: a definition and systematic approach to component interventions. Dis Manage Health Outcomes 2003;11(8):477-88.
62. Haskell W, Alderman E, Fair J, Maron D, Mackey S, et al. Effects of intensive multiple risk factor reduction on coronary atherosclerosis and clinical cardiac events in men and women with coronary artery disease. The Stanford coronary risk intervention project (SCRIP). Circulation 1994;89:975-90.
63. Gilmer T, PJ OC. Cost effectiveness of diabetes mellitus management programs. A health plan perspective. Dis Manage Health Outcomes 2003;11(7):439-53.
64. Wagner E, Sandhu N, Newton K, McCulloch D, Ramsey S, Grothaus L. Effect of imporved glycemic control on health care costs and utilization. JAMA 2001;285:182-9.
65. Testa M, Simonson D. Health economic benefits and quality of life during improved glycemic control in patients with type 2 diabetes mellitus. JAMA 1998;280:1490-6.
66. VHA's Mission, Vision, Values, Strategies. Dept. of Veterans Affairs, 2005. (Accessed May 30th, 2006, at http://vaww.visn5.med.va.gov/resources/career_dev/memo_sep05.pdf.)
67. Stout RL, Wirtz PW, Carbonari JP, Boca FKD. Ensuring balanced distribution of prognostic factors in treatment outcome research. J Stud Alcohol Suppl 1994;12:70-5.

68. Bartholomew LK, Parcel GS, Kok G, Gottlieb NH. Theories in health education and promotion. In: Bartholomew LK, Parcel GS, Kok G, Gottlieb NH, eds. Intervention mapping Designing theory-and evidece-based health promotion programs. New York, NY: McGraw-Hill; 2001:96-100.
69. Berkman L, Glass T. Social Integration, Social Networks, Social Support, and Health. In: Berkman L, Kawachi I, eds. Social Epidemiology. New York: Oxford Press; 2000.
70. Heaney C, Israel B. Social networks and social support. In: Glanz K, Rimer B, Lewis F, eds. Health Behavior and Health Education Theory, research and practice. 3rd ed. San Francisco: John Wiley & Sons, Inc.; 2002:185-206.
71. Minkler M, Wallerstein N. Improving health through community organization and community building. In: Glanz K, Rimer B, eds. Health Behavior and Health Education: theory, research, and practice. 3rd ed. San Francisco: John Wiley & Sons, Inc.; 2002.
72. Mensing C, Boucher J, Cypress M. National standards for diabetes self-management education. Diabetes Care 2002;25:s140-s7.
73. Viner RM, Christie D, Taylor V, Hey S. Motivational/solution-focused intervention improves HbA1c in adolescents with type 1 diabetes: a pilot study. Diabet Med 2003;20(9):739-42.
74. Channon S, Smith VJ, Gregory JW. A pilot study of motivational interviewing in adolescents with diabetes. Arch Dis Child 2003;88(8):680-3.
75. Motivational Interviewing. Mid-Atlantic Addiction Technology Transfer Center, 2006. (Accessed June 12, 2006, at
76. Prochaska JO, DiClemente CC. Stages and processes of self-change of smoking: Toward an intergrative model of change. Journal of Consulting and Clinical Psychology 1983;51(3):390-5.
77. Measurement Excellence and Training Resource Information Center. Critical review of Medical Outcomes Study 36-Item Short-Form Health Survey (SF-36). 2003. (Accessed January 2nd., 2006, at http://www.measurementexperts.org//instrument/instrument_reviews.asp?detail=54.)
78. Kazis LE, Lee A, Spiro III A, et al. Measurement Comparisons of the Medical Outcomes Study and Veterans SF-36® Health Survey. HEALTH CARE FINANCING REVIEW 2004;25:43-58.
79. Ahroni J, Boyko E. Responsiveness of the SF-36 among veterans with diabetes mellitus. J Diabetes Complications 2000;14(1):31-9.
80. Luscombe F. Health-related quality of life measurement in type 2 diabetes. Value Health 2000;3 Suppl1:15-28.
81. Boyer JG EJ. The development of an instrument for assessing the quality of life of people with diabetes: diabetes-39. Med Care 1997;35(5):440-53.
82. Measurement Excellence and Training Resource Information Center. Critical review of Short Form-36 for Veterans (SF-36V). 2002. (Accessed January 02., 2006, at http://www.measurementexperts.org//instrument/instrument_reviews.asp?detail=35.)
83. Smith MW, Barnett PG, Phibbs CS, Wagner TH, Yu W. Micro-cost methods for determining VA healthcare costs. Menlo Park, CA: Health Economics Resource Center U.S. Dept. of Veterans Affairs; 2005.
84. Bhandari A, Wagner T. Self-Reported Utilization of Health Care Services: Improving Measurement and Accuracy. Med Care Res Rev 2006;63(2):217-35.

85. American Medical Association. Medicare RBRVS: A Physicians' Guide. Chicago, IL: American Medical Association; 2001.
86. Cohen SB, DiGaetano R, Goksel H. MEPS Methodology Report No. 5, Estimation Procedures in the 1996 Medical Expenditure Panel Survey, Household Component. Rockville, MD: Agency for Healthcare Research and Quality; 1999.
87. Agency for Healthcare Resarch and Quality. Data Documentation, MEPS HC-012: 1996 Full Year Consolidated Data File. Rockville, MD: Agency for Healthcare Resarch and Quality; 2000.
88. Williams GC, Freedman ZR, Deci EL. Supporting autonomy to motivate patients with diabetes for glucose control. Diabetes Care 1998;21(10):1644-51.
89. Heisler M, Vijan S, Anderson RM, Ubel PA, Bernstein SJ, Hofer TP. When Do Patients and Their Physicians Agree on Diabetes Treatment Goals and Strategies, and What Difference Does It Make? Journal of General Internal Medicine 2003;18(11):893-902.
90. Toobert DJ, Hampson SE, Glasgow RE. The summary of diabetes self-care activities measure: results from 7 studies and a revised scale. Diabetes Care 2000;23(7):943-50.
91. Perceived Competence Scales. In: University of Rochester; 2004.
92. Bandura A. Self-efficacy: the exercise of control. New York, NY: Freeman; 1997.
93. Glasgow RE, Toobert DJ, Hampson SE, Brown JE, Lewinsohn PM, Donnelly J. Improving self-care among older patients with type II diabetes: the "Sixty Something." Study. Patient Educ Couns 1992;19:61-74.
94. Glasgow RE, Strycker LA, Toobert DJ, Eakin E. A social-ecologic approach to assessing support for disease self-management: the Chronic Illness Resources Survey. J Behav Med 2000;23(6):559-83.
95. Radloff LS. The CES-D scale: A self-report depression scale for research in the general population. Applied Psychological Measurement 1977;1:385-401.
96. Beck AT, Ward CH, Medelson M, Mock J, Erbaugh J. An inventor of measuring depression. Arch Gen Psychiatry 1961;4:561-71.
97. Zung WWK. A self-rating depression scale. Arch Gen Psychiatry 1965;12:63-70.
98. Yin-Ling IW. Measurement properties of the center for epidemiologic studies - depression scale in a homeless population. Psychol Assess 2000;12:69-76.
99. Charlson ME, Pompei P, Ales KL, MacKenzie CR. A new method of classifying prognostic comorbidity in longitudinal studies: Development and validation. J Chron Dis 1987;40:373-83.
100. Veterans Health Administration. Decision Support Office. Decision Support System. Veterans Health Administration, 2005. (Accessed January 10th, 2006, at http:/vaww.dss.med.va.gov/.)
101. Liang K, Zeger S. Longitudinal data analysis using generalized linear models. Biometrika 1986;73:13-22.
102. Zeger S, Liang K. Longitudinal data analysis for discrete and continuous outcomes. Biometrics 1986;42:121-30.
103. Dobson AJ. An Introduction to Genearlized Linear Models. London: Chapman & Hall; 1990.
104. Goldstein H, Rasbach J, Plewis, I, al. e. A User's Guide to MLWIN. 1 ed. London, UK: Multilevel Models Project, Institute of Education, University of London; 1998.

105. Austin PC, T JV, Alter DA. Comparing hierarchical modeling with traditional logistic regression analysis among patients hospitalized with acute myocardial infarction: Should we be analyzing cardiovascular outcomes data differently? American Heart Journal 2003;145(1):27.
106. Baron RM, Kenny DA. The Moderator-Mediator Variable Distinction in Social Psychological Research: Conceptual, Strategic, and Statistical Considerations. Journal of Personality and Social Psychology 1986;51:1173-82.
107. Littell R, Milliken G, Stroup W, Wolfinger R. SAS system for mixed models. Cary, NC: SAS Institute; 1996.
108. Sobel ME. Asymptotic confidence intervals for indirect effects in structural equation models. In: Leinhardt S, ed. Sociological Methodology. Washingtion, D.C.: American Sociological Association; 1982:290-312.
109. Krull JL, MacKinnon DP. Multilevel mediation modeling in group-based intervention studies. Evaluation Review 1999;23:418-44.
110. Little RJA, Rubin DB. Statistical Analysis with Missing Data. New York: John Wiley; 1987.
111. Schafer JL. Analysis of incomplete multivariate data. New York: Chapman & Hall; 1997.
112. Rubin DB. Multiple imputation for nonresponse in surveys. New York: Wiley; 1987.
113. Hsieh FY, Lavori PW, Cohen HJ, Fuessner JR. An overview of variance inflation factors for sample-size calculation. Palo Alto, CA: Dept. of Veterans Affairs Cooperative Studies Program; 2003 September.
114. Fleiss J, Cuzick J. The reliability of dichotomous judgments: Unequal numbers of judges per subject. Applied Psychological Measurement 1979;3:537-42.
115. Zou G, Donner A. Confidence interval estimation of the intraclass correlation coefficient for binary outcome data. Biometrics 2004;60:807-11.
116. Ho P, Maynard C, Starks H, Sun H, Sloan K, Sales A. Outcomes in patients with coronary heart disease who do not undergo lipid testing. Am J Cardiol 2003;91:986-8.
117. International Committee of Medical Journal Editors. Uniform requirements for manuscripts submited to biomedical journals. JAMA 1997;277:927-34.
118. Bauer MS, Williford WO, Dawson EE, et al. Principles of effectiveness trials and their implementation in VA Cooperative Study #430: 'Reducing the efficacy-effectiveness gap in bipolar disorder'. Journal of Affective Disorders 2001;67:61-78.
119. Bauer MS, McBride L, Williford WO, Glick H, Kinosian B, et al. Collaborative care for bipolar disorder, part I: intervention and implementation in a multi-site randomized effectiveness trial. Psychiatric Services 2006:In press.
120. Bauer M, McBride L. Structured group psychotherapy for bipolar disorder. The life goals program. 2nd ed. New York: Springer Publishing company, Inc; 2003.

Table of Major Study Events

November 14, 2007	Initial study protocol approval by IRB
April 1, 2008	Start of funding for IAB 06-269.
April 30, 2008	Protocol modification – addition of data collection instrument (IIEF-5 questionnaire)
July 1, 2008	Beginning of study recruitment Providence site.
July 15, 2008	Protocol modification – addition of data collection instrument (Medication concordance survey)
August 13, 2008	Protocol modification – addition of gas cards for study visits
September 1, 2008	Beginning of study recruitment West Haven site.
September 23, 2008	Protocol modification — refinement of HIPAA form to conform current standards
October 8th, 2008	Protocol modification – Redefinition of study endpoint and power calculation according to the DSMB suggestions
October 21 and 23, 2008	Protocol modification — refinement of patient recruitment to include stamped return envelope for best times to call and include a basic information package about diabetes self care for all study participants
November 12, 2008	Continuing review approved
December 1, 2008	First CoMB report
July 13, 2009	Mid-Year Progress Report
August 19, 2009	Request to allow subjects to receive pharmacist- care during the study period if so prescribed by their primary care physician, to reflect true "usual or standard are". (approved 8/20/09)
August 24, 2009	Modification request: Addition of VA Pacific Islands Health Care System (VA Honolulu and American Samoa CBOC) as additional sites (approved 8/18/09)
September 22, 2009	Modification request: Addition of echocardiogram for PVAMC site (approved 10/14/09)
October 14, 2009	Continuing review approved (10/14/09 – 10/13/10)
December 1, 2009	Annual DSMB report (accepted 1/21/10)
December 22, 2009	Modification to add an anonymous survey to the intervention group upon study completion. (approved 12/23/09)
February 2, 2010	Beginning of study recruitment at the Honolulu site
July 14, 2010	Mid-Year Progress Report to DSMB (accepted 8/11/10)
